# Therapeutic targeting de novo purine biosynthesis driven by β-catenin-dependent PPAT upregulation in hepatoblastoma

**DOI:** 10.1038/s41419-025-07502-6

**Published:** 2025-03-17

**Authors:** Ming Ding, Chunshuang Ma, Yanyan Lin, Houshun Fang, Yan Xu, Shuxuan Wang, Yao Chen, Jiquan Zhou, Hongxiang Gao, Yuhua Shan, Liyuan Yang, Huiying Sun, Yabin Tang, Xiaoyu Wu, Liang Zhu, Liang Zheng, Yehuda G. Assaraf, Bin-Bing S. Zhou, Song Gu, Hui Li

**Affiliations:** 1 Pediatric Translational Medicine Institute, Key Laboratory of Pediatric Hematology & Oncology Ministry of Health, Department of General Surgery, Shanghai Children’s Medical Center, Shanghai Jiao Tong University School of Medicine, Shanghai, 200127 China; 2 Fujian Children’s Hospital, Fujian Branch of Shanghai Children’s Medical Center Affiliated to Shanghai Jiao Tong University School of Medicine, Fuzhou, 350014 China; 3 Department of Pharmacology and Chemical Biology, School of Basic Medicine and Shanghai Collaborative Innovation Center for Translational Medicine Ministry of Education, Shanghai Jiao Tong University School of Medicine, Shanghai, 200025 China; 4 The Fred Wyszkowski Cancer Research Laboratory, Faculty of Biology, Technion-Israel Institute of Technology, Haifa, 3200003 Israel

**Keywords:** Paediatric cancer, Liver cancer, Cancer metabolism, Mechanisms of disease

## Abstract

De novo purine biosynthesis (DNPS) was previously shown to be aberrantly activated in many cancers. However, the activity of DNPS pathway and its underlying regulatory mechanism in hepatoblastoma (HB) remain poorly understood. Herein, we discovered that the expression of PPAT, the rate-limiting enzyme in DNPS, was markedly upregulated in HB, leading to an augmented purine flux via DNPS, thereby promoting both HB cell proliferation and migration. Furthermore, we found that activated mutant β-catenin, a dominant driver of HB, transcriptionally activated *PPAT* expression, hence stimulating DNPS and constituting a druggable metabolic vulnerability in HB. Consistently, pharmacological targeting using a DNPS inhibitor lometrexol or genetic repressing the enhanced DNPS markedly blocked HB progression in vitro and in vivo. Our findings suggest that HB patients harboring activated β-catenin mutations and consequent DNPS upregulation, may be treated efficaciously with DNPS enzyme inhibitors like lometrexol. These novel findings bear major therapeutic implications for targeted precision medicine of HB.

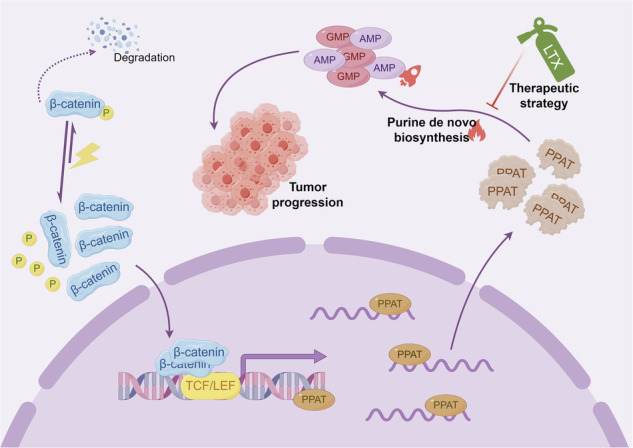

## Introduction

Hepatoblastoma (HB) is a common embryonal malignant liver cancer occurring in children, accounting for approximately 80% of pediatric liver neoplasms and originating from aberrant stem cells or hepatic progenitor cells [1]. The prognosis of HB has currently been improved following the combination treatment of surgery and neoadjuvant chemotherapy [2, 3]. However, advanced-stage patients with metastatic and/or chemotherapy-resistant HB, do not respond to standard treatment, and their 5-year survival rate is less than 40% [4–6]. Thus, deciphering the molecular mechanisms underlying HB progression is of urgent and paramount importance to enlighten new therapeutic strategies.

HB is a Wnt/β-catenin-driven pediatric liver cancer in which 50–90% of patients carry activating mutations in *CTNNB1*, the gene encoding for β-catenin [7, 8]. *CTNNB1* activating mutations enhance β-catenin protein stability and facilitate its translocation to and accumulation in the nucleus, where β-catenin binds to the TCF/LEF transcription factors and subsequently drives transactivation of many target genes, thereby promoting tumor initiation and progression [9]. Metabolic alterations represent a hallmark of cancer cells, while biosynthesis and utilization of nucleotides is a fundamental metabolic requirement in various cancer types with distinct genetic backgrounds [10, 11]. Purine nucleotide biosynthesis consists of de novo and salvage pathways. De novo purine biosynthesis (DNPS) is initiated with 5-phosphoribosyl-1-pyrophosphate (PRPP) which is then incorporated into nucleobases onto the ribose ring framework through the catalytic activity of six DNPS enzymes [12]. On the other hand, the purine salvage pathway involves recycling of available nucleobases and nucleosides to supplement the intracellular pool of nucleotides [12]. In comparison to the salvage pathway, DNPS is an energy-consuming pathway, hence its activity is typically low or even shutdown in non-proliferating cells [13, 14]. However, DNPS was found to undergo activation, which is crucial for cancer cell survival, proliferation and drug resistance, hence constituting a promising therapeutic target in a variety of cancers [15–19]. In small cell lung cancer, phosphoribosyl pyrophosphate amidotransferase (PPAT), a key enzyme of DNPS, can synergize with glutaminase (GLS) to determine the metabolic fate of glutamine within the cell, thereby sustaining DNA replication and tumor cell proliferation [20]. In breast cancer, phosphoribosylaminoimidazole carboxylase and phosphoribosylaminoimidazolesuccinocarboxamide synthase (PAICS), a bifunctional enzyme in DNPS, modulates the activity of estrogen receptor α (Erα) and confers tamoxifen resistance upon breast cancer cells [21]. Moreover, tumor cells also activate the mammalian target of rapamycin complex 1 (mTORC1) signaling pathway to promote purine biosynthesis [22]. Moreover, brain tumor initiating cells and leukemia cells exhibit dependence on DNPS and thus inhibition of DNPS was found to abrogate tumor formation both in vitro and in vivo [23, 24]. However, the activity of DNPS and its molecular mechanism driving HB progression remain poorly understood.

As a step towards this end, we herein demonstrated that the PPAT expression was upregulated and the DNPS activity was significantly enhanced in HB. PPAT was found essential for HB progression as evidenced by enhanced cell proliferation, cell migration and tumor progression. We further found that β-catenin could translationally activated PPAT expression and stimulate DNPS pathway, which is essential for HB progression. Importantly, we demonstrated that targeted pharmacological inhibition of DNPS, blocked HB cell proliferation and tumor progression, thereby offering a potential therapeutic strategy for HB treatment.

## Materials and methods

### Sample obtainment and cell culture

Paired HB tumor samples and adjacent normal tissues were obtained from patients treated at Shanghai Children’s Medical Center (SCMC). All the patients have signed informed consent. The Institutional Review Board of SCMC approved the current study.

The HuH6, HepG2, QSG7701 and HEK-293T cell lines were cultured in DMEM (Gibco) supplemented with 10% fetal bovine serum (FBS, Sigma) and 1% penicillin-streptomycin (NCM Biotech) at 37 °C in 5% CO_2_. Hypoxanthine (HX, 100 µM) was added to the growth medium of PPAT knockout cells. Cell lines were authenticated via short tandem repeat (STR) profiling and tested for mycoplasma contamination on a regular basis.

### RNA extraction and reverse transcription quantitative PCR analysis

Total RNA was extracted using FastPure Cell/Tissue Total RNA Isolation Kit V2 (Cat. RC112, Vazyme) and reverse transcribed (RT) to cDNA using Evo M-MLV RT Mix Kit with gDNA Clean for qPCR Ver.2 (Cat. AG11728, Accurate Biology). Quantitative PCR (qPCR) reactions were carried out with SYBR Green Premix Pro Taq HS qPCR Tracking Kit (Cat. AG11701, Accurate Biology). Relative mRNA expression levels were normalized to *ACTB*.

Primers used in qPCR included: *PPAT*, F-GATCACTCTGGGACTCGTGG, R-CAAGACCCATTCCCTTGTGTG; *CTNNB1*, F-GCCGGCTATTGTAGAAGCTG, R-GAGTCCCAAGGAGACCTTCC; *AXIN2*, F-TACACTCCTTATTGGGCGATCA, R-TTGGCTACTCGTAAAGTTTTGGT; *ACTB*, F-CATCCGCAAAGACCTGTACG, R-CCTGCTTGCTGATCCACATC.

### Western blot analysis

Patient samples or cultured HB cells were homogenized in lysis buffer and the proteins were resolved by SDS-PAGE. The primary antibodies included: PPAT (Cat. TA504769, OriGene), β-catenin (Cat. 8480, CST), γ-H2AX (S139) (Cat. ab81299, Abcam), H2AX (Cat. 7631, CST), p-CHK2 (T68) (Cat. 2661, CST), CHK2 (Cat. 3428-1, Epitomics), PARP (Cat. 9542, CST), cleaved PARP (Cat. 5625, CST), Axin2 (Cat. 20540-1-AP, Proteintech), and β-actin (Cat. 66009-1-Ig, Proteintech). The secondary antibodies included: HRP-conjugated Goat anti-Rabbit/Mouse IgG (Cat. HA1001/HA1006, HUABIO), and IRDye 800CW Goat anti-Rabbit/Mouse IgG (Cat. 926-32211/926-32210, LI-COR). Immunoblots were detected by Chemi Doc MP Imaging System (Bio-Rad).

### Immunohistochemistry analysis

Paired HB tumor samples and adjacent normal tissues obtained from patients or subcutaneous HB tumors harvested from xenograft mouse models were fixed overnight with 4% paraformaldehyde, dehydrated with gradient ethanol, and embedded in paraffin. Sections were de-paraffinized and re-hydrated, followed by Hematoxylin and Eosin (H&E) staining or immunohistochemistry (IHC) staining. The antibodies used in IHC staining included: PPAT (Cat. TA504769, OriGene), β-catenin (Cat. 8480, CST) and Perilipin-1 (Cat. 9349, CST). The sections were finally scanned using KF-PRO-120 Digital Pathology Slide Scanner (KFBIO).

### Lentivirus production and cell transfection

CRISPR-Cas9 system was used to knockout genes in HB cells. Single-guide RNA was designed based on information available at the Zhang’s lab website and was cloned into a lentiCRISPR v2 vector (Addgene) following their protocol. The sequences targeting *PPAT and HPRT1* were 5′-CACAGGGGTCAGGAGAGTGC-3′ and 5′-CATACCTAATCATTATGCTG-3′, respectively. The sequence of the non-targeting control was 5′-GGATACTTCTTCGAACGTTT-3′. Short hairpin RNA (shRNA) was used to knockdown genes in HB cells. shRNA was designed based on information available at the Sigma website and was cloned into a pLKO IPTG 3xLacO vector (Sigma) following their protocol. The sequence targeting *CTNNB1* was 5′-TTGTTATCAGAGGACTAAATA-3′. The sequence of the non-targeting control was 5′-GCGCGATAGCGCTAATAATTT-3′. Human *PPAT* coding region was cloned into pGV303 vector (Genechem) and confirmed by Sanger sequencing.

Lentivirus was produced in HEK-293T cells via co-transfecting constructed plasmid, psPAX2 and pMD2.G using jetPRIME (Polyplus). The supernatant of cell culture medium was harvested 48 h and 72 h after transfection, following which the supernatant was concentrated in a 100 kDa Amicon Ultra Centrifugal Filter (Millipore). Lentivirus was then used to transfect HB cells.

### Cell proliferation and cell viability assay

HB cells were seeded in 96-well plates and cell viability was detected by CellTiter-Glo Luminescent Cell Viability Assay (Cat. G7573, Promega) following the manufacturer’s instructions, the luminescence was then detected using a Synergy H1 Multimode Microplate Reader (BioTek). In proliferation assay, 2000 cells were seeded in each well of 96-well plates and cell viability was assessed once a day for 5 days. In viability assay, 4000 cells were seeded in each well of 96-well plates. Following an overnight incubation, cells were treated with increasing concentrations of drugs for 72 h and cell viability was then assessed.

### Apoptosis and cell cycle analysis

Cell apoptosis was determined with Annexin V-APC/PI Apoptosis Kit (Cat. E-CK-A217, Elabscience) following the manufacturer’s instructions. In brief, HB cells were harvested and washed twice with ice-cold PBS, then stained with Annexin V-APC and propidium iodide (PI) for 25 min, and finally analyzed by flow cytometry using a BD FACSCanto Flow Cytometer (BD Biosciences).

Cell cycle was determined with Click-iT EdU Alexa Fluor 647 Flow Cytometry Assay Kit (Cat. C10419, Invitrogen), following the manufacturer’s instructions. In brief, HB cells were incubated with EdU for 30 min, fixed for 15 min, permeabilized for 15 min and incubated with freshly prepared Click-iT reaction cocktail for 30 min, then stained with PI for 10 min and finally analyzed by flow cytometry using a BD FACSCanto Flow Cytometer (BD Biosciences).

### Colony formation assay

HB cells were seeded in 6-well plates (200 cells/well) and cultured for 2 weeks at 37 °C in 5% CO_2_. Cells were then harvested, washed twice with ice-cold PBS, fixed with 4% paraformaldehyde for 20 min and stained with Crystal Violet (Cat. E607309, Sangon Biotech) for 2 h. Cell colony images were finally captured by optical microscopy (Leica) and analyzed with ImageJ software.

### Transwell migration assay

HB cells (1 × 10^4^ HuH6 or 5 × 10^4^ HepG2 cells/well) were suspended in 200 μL serum-free DMEM and seeded in the upper part of a transwell chamber (8 μm pore size, Costar), and 500 μL DMEM containing 10% FBS was placed in the lower part of the chamber. Following incubation for 24 h, the transwell chambers were fixed with 4% paraformaldehyde for 20 min, and then stained with Crystal Violet (Cat. E607309, Sangon Biotech) for 2 h. The cells on the top surface of the chamber were gently removed with a cotton swab, and the migrating cells located at the bottom surface were captured by optical microscopy (Leica) and analyzed with ImageJ software.

### Wound healing assay

HB cells were seeded in 6-well plates at a density of 90% confluence. Following cell attachment, the cell monolayer was scratched with a 200 μL pipette tip, then washed once with PBS. The cells were incubated in growth medium at 37 °C in 5% CO_2_. Images of the scratched area were captured by optical microscopy (Leica) at time 0 and 24 h after scratching and analyzed using an ImageJ software.

### Xenograft mouse model

Animal studies were approved by the Institutional Animal Care and Use Committee (IACUC) of SCMC. Six-week-old female nude mice (GemPharmatech) were used in the current study. A total of 5 × 10^6^ indicated HuH6 cells were subcutaneously injected into the right flank of each mouse. Tumor volumes and mice body weights were measured twice a week, and tumor volumes were calculated using the following formula: Volume (mm^3^) = Length (mm) × Width^2^ (mm^2^) × 0.5. After approximately 1 week, lometrexol (LTX, 40 mg/kg) was intraperitoneally injected to mice once a day for 7 days. Mice were sacrificed when tumors reached a volume of 1000 mm^3^, the tumors were then harvested, and tumor weights were determined.

### Dual-luciferase reporter assay

The human *PPAT* promoter region containing 6 wild type (WT) β-catenin binding sites (−1721 to −1386 from the *PPAT* transcription start site) was cloned into pGL4.10 vector (Promega) and confirmed by Sanger sequencing. The human *PPAT* promoter region with the mutated β-catenin binding sites was synthesized and cloned into pGL4.10 vector (Promega).

Dual-luciferase reporter assay was preformed using Dual-Luciferase Reporter Assay System (Cat. E1910, Promega) following the manufacturer’s instructions. In brief, HEK-293T cells were co-transfected with the constructed luciferase reporter plasmid, control *Renilla* luciferase vector and β-catenin-expressing plasmid. HB cells were co-transfected with either the constructed luciferase reporter plasmid or Super 8x TOPFlash/FOPFlash (Addgene) and control *Renilla* luciferase vector. After culturing for 24 h, cells were lysed with passive lysis buffer and firefly luciferase activities were first measured, followed by *Renilla* luciferase activity measurement, using a Synergy H1 Multimode Microplate Reader (BioTek). Relative firefly luciferase activities were normalized to *Renilla* luciferase activities.

### Chromatin immunoprecipitation quantitative PCR assay

Chromatin immunoprecipitation quantitative PCR (ChIP-qPCR) assay was performed using SimpleChIP Enzymatic Chromatin IP Kit (Cat. 9003, CST) following the manufacturer’s instructions. In brief, HuH6 cells were cross-linked with 1% formaldehyde for 10 min and the reaction was then terminated by glycine. The extracted chromatin was digested and fragmented to around 500 bp, and then immunoprecipitated using β-catenin antibody (Cat. 8480, CST) or normal rabbit IgG (Cat. sc-3888, Santa Cruz) with protein G magnetic beads. The complexes were finally uncross-linked to obtain pure DNA fragments for qPCR. Primers for the *PPAT* promoter region with β-catenin binding sites used in qPCR were as follows: Forward-ACACACCCCAAGCTATCGTG, Reverse-GGGCGTGTGTGTAAGTAGT.

### Isotope tracing assay

HuH6 cells were seeded in 100 mm dishes and cultured until 80% confluency. The cells were washed twice with respective metabolite-free medium, then medium containing 0.4 mM ^13^C_2_, ^15^N-Glycine (Cat. 489522, Sigma) was added 4 h before cell harvesting or medium containing 4 mM ^15^N-Glutamine (Cat. NLM-557, Cambridge Isotope Laboratories) was added 30 min before cell harvesting.

To collect metabolites, cells were washed three times with ice-cold PBS and metabolites were extracted using 80% methanol (pre-cooled to −80 °C). After centrifugation at 14,000 × *g* for 20 min at 4 °C, the metabolite-containing supernatant was dried under a stream of nitrogen gas and stored at −80 °C until analyzed by liquid chromatography-mass spectrometry (LC–MS) at the Translational Medicine Collaborative Innovation Center of Shanghai Jiao Tong University School of Medicine.

### Statistical analysis

GraphPad Prism 8 software was used for statistical analysis. Results were presented as means ± SD, and statistical significance was calculated by Student’s *t* test or Fisher’s exact test, *p* < 0.05 represented a statistically significant result.

Gene set enrichment analysis (GSEA) was performed with GSEA v4.3.3 for Windows (https://www.gsea-msigdb.org/gsea/index.jsp) using GSEAPreranked mode, default parameters and the gene set database h.all.v2024.1 and c5.go.bp.v2024.1 from Human MSigDB (https://www.gsea-msigdb.org/gsea/msigdb/index.jsp).

Kaplan–Meier survival analysis was performed with Kaplan–Meier Plotter online tool (https://www.kmplot.com/analysis/) using Pan-cancer RNA-seq mode (selecting Liver hepatocellular carcinoma only), default parameters (auto select best cutoff—all, no restriction to subtypes, no restriction on cellular content).

## Results

### Elevated expression of PPAT serves as a potential diagnostic biomarker for HB

**Fig. 1:**
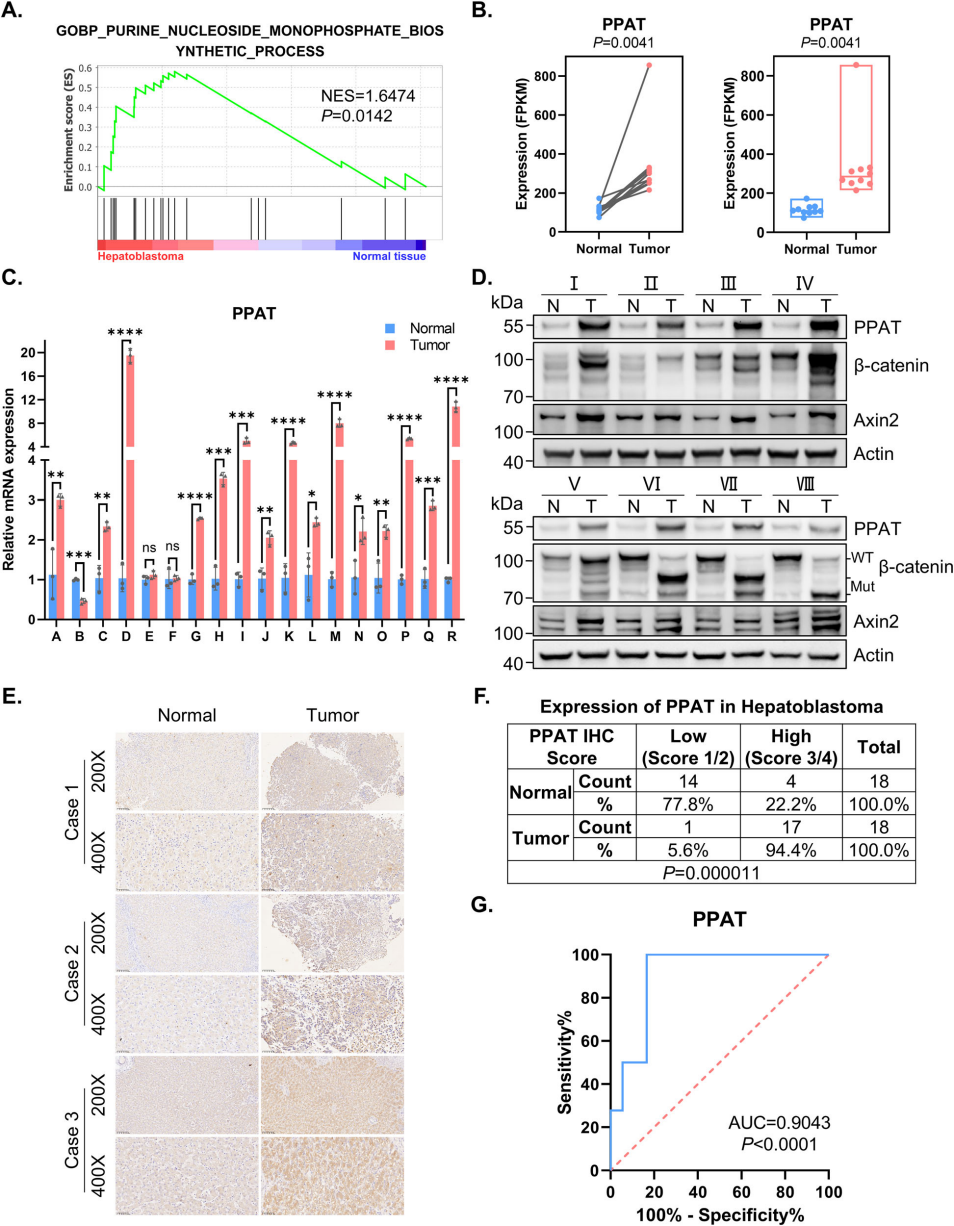
The de novo purine biosynthesis pathway is upregulated in HB and is associated with poor prognosis. **A** GSEA evaluating gene signature of purine nucleoside monophosphate biosynthesis in paired HB and normal tissues (*n* = 10). NES normalized enrichment score. **B** The mRNA level of PPAT in paired HB and normal tissues (*n* = 10) determined by RNA-seq (paired *t*-test). **C** The mRNA level of PPAT in paired HB and normal tissues (*n* = 18) validated by RT-qPCR (paired *t*-test). *p value < 0.05, **p value < 0.01, ***p value < 0.001, and ****p value < 0.0001. This statistical significance grading was kept constant throughout the entire paper. **D** The protein levels of PPAT, β-catenin and Axin2 in paired HB and normal tissues (*n* = 8) were validated by WB analysis. **E** Representative IHC analysis of PPAT in paired HB and normal tissues. **F** Fisher’s exact test of PPAT expression level in paired HB and normal tissues (*n* = 18). The level was scored by grading criteria based on the percentage of cells with various IHC staining intensity. **G** ROC curve for using PPAT as a diagnostic biomarker.

To explore the potential roles of purine biosynthesis in HB, we initially examined the expression of genes associated with purine biosynthesis by using RNA sequencing (RNA-seq) analysis in paired HB tumor samples and adjacent normal liver tissues. Gene set enrichment analysis (GSEA) revealed a significant enrichment of upregulated genes mediating purine biosynthesis in HB specimens, compared to paired normal tissues (Fig. 1A and Table S1). This analysis of RNA-seq data from paired HB patient samples identified significantly elevated expression of major enzymes mediating DNPS, including PPAT, glycinamide ribonucleotide transformylase (GART), phosphoribosylformylglycinamidine synthase (PFAS), PAICS, adenylosuccinate lyase (ADSL) and aminoimidazole carboxamide ribonucleotide transformylase (ATIC) (Figs. 1B and S1A). In contrast, we did not obtain similar results with genes encoding for enzymes in purine salvage pathway (Fig. S1B). Consistently, we also observed increased PPAT expression in HB tumor samples in two independently published HB datasets (GSE132219 and GSE104766) (Fig. S1C).

To validate our findings based upon RNA-seq data, we performed reverse transcription quantitative PCR (RT-qPCR) in paired HB specimens and adjacent normal tissues, confirming the increased PPAT expression in 15 of 18 HB specimens, unchanged PPAT expression in two specimens and decreased PPAT expression in one specimen (Fig. 1C). Furthermore, this markedly increased PPAT expression was also noted in human HB cell lines (HuH6 and HepG2) when compared to a normal hepatic cell line (QSG7701) (Fig. S1D). Expectedly, Western blot (WB) analysis revealed that PPAT protein levels were consistently elevated in HB specimens compared to adjacent normal tissues (Fig. 1D and Table S2). We also performed IHC analysis on 18 paired HB patient samples (Fig. 1E); most HB tissues (94.4%) exhibited a high score of positive PPAT staining, compared with adjacent normal tissues (22.2%, *p* < 0.001) (Fig. 1F).

Given the increased PPAT expression in HB patient samples, we further investigated the diagnostic value of PPAT in distinguishing HB tissues from normal tissues. The receiver operating characteristic (ROC) curve demonstrated the diagnostic value of *PPAT* expression (AUC = 0.9043, *p* < 0.0001) (Fig. 1G). Furthermore, given the lack of compelling publicly available HB datasets containing adequate sample size, we undertook a putative surrogate analysis using liver cancer datasets. This Kaplan–Meier survival analysis based on a large public liver cancer dataset indicated that high expression levels of PPAT, along with other five DNPS genes, were correlated with poor prognosis (Fig. S1E, F). Clearly, this putative surrogate analysis warrants an equivalent survival analysis with genuine HB datasets when such a proper size cohort becomes available. Collectively, these results suggest that PPAT levels could potentially serve as a biomarker for diagnosis and prediction of the prognosis of HB patients.

### PPAT is required for HB cell proliferation

**Fig. 2:**
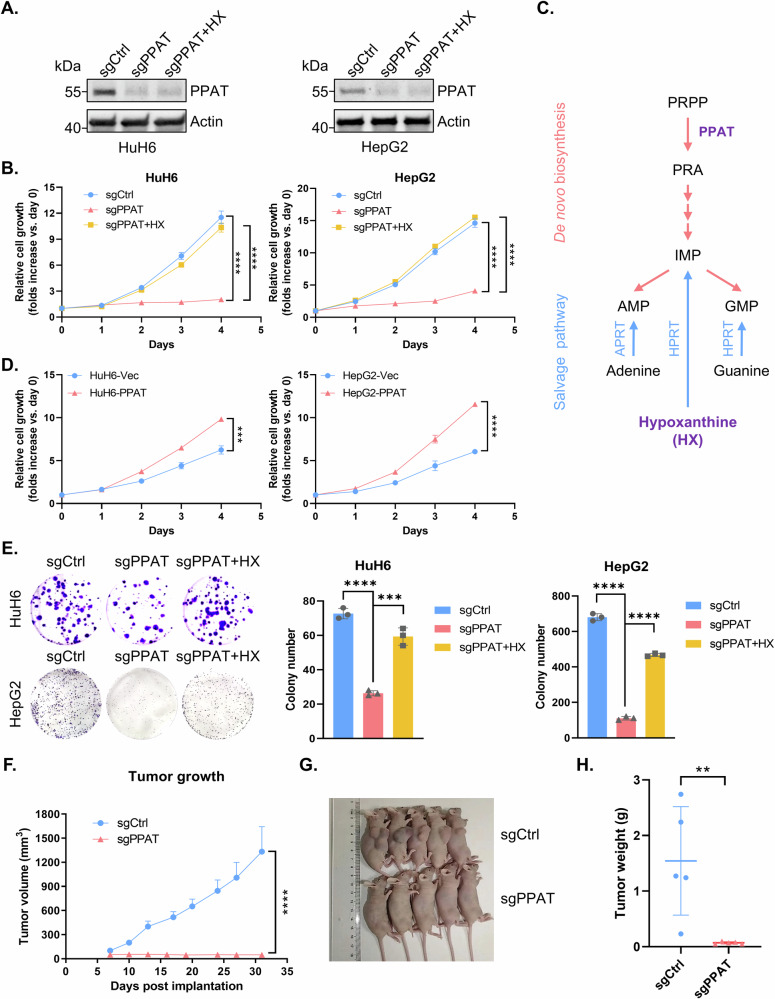
PPAT, the rate-limiting de novo purine biosynthesis enzyme, is essential for HB cell proliferation. **A** The protein level of PPAT was detected by WB analysis in non-targeting control and PPAT knockout HuH6 or HepG2 cells with or without supplementation of 100 μM HX in the growth medium. **B** Growth curves were generated for non-targeting control and PPAT knockout HuH6 or HepG2 cells with or without supplementation of 100 μM HX. Each point was assayed in quadruplicates/hexaplicates (unpaired *t*-test). **C** A scheme of de novo and salvage purine biosynthesis pathway. **D** Growth curves were generated for vector and PPAT overexpressing HuH6 or HepG2 cells. Each point was assayed in triplicates (unpaired *t*-test). **E** Non-targeting control and PPAT knockout HuH6 or HepG2 cells with or without supplementation of 100 μM HX, were subjected to colony formation assay (left) and the results were quantified (right), *n* = 3 (unpaired *t*-test). **F** PPAT knockout effectively suppressed HB tumor growth in nude mice subcutaneously implanted with indicated HuH6 cells. Tumor volume was monitored twice a week (unpaired *t*-test). Tumor size (**G**) and weight (**H**) at the end point (unpaired *t*-test).

To substantiate the involvement of PPAT in HB progression, we employed CRISPR-Cas9 to knockout PPAT in the human HB cell lines HuH6 and HepG2 and validated PPAT protein levels by WB analysis (Fig. 2A). A subsequent cell proliferation assay revealed a marked tumor cell growth inhibition following PPAT knockout (Fig. 2B). Importantly, supplementing the growth medium with hypoxanthine (HX), a key nucleobase in the purine salvage pathway, almost fully restored impaired tumor cell proliferation caused by PPAT knockout (Figs. 2B, C and S2A). In contrast, knocking out hypoxanthine phosphoribosyltransferase 1 (HPRT1), a crucial enzyme involved in the purine salvage pathway, had no impact on tumor cell proliferation (Fig. S2B), indicating the specific dependence of HB cells on DNPS for cell proliferation. Remarkably, overexpressing PPAT in either HuH6 or HepG2 cells significantly boosted tumor cell growth (Fig. 2D). Furthermore, PPAT knockout substantially inhibited colony formation in both HuH6 and HepG2 cells, which was readily restored by supplementation of the growth medium with HX (Figs. 2E and S2C). To further ascertain the potential role of PPAT on HB tumor growth in vivo, we conducted subcutaneous HB tumor xenograft transplantation using nude mice. Remarkably, our results demonstrated that depletion of PPAT completely abolished HB tumor growth in vivo (Fig. 2F–H). In conclusion, these results establish the central role of PPAT in HB progression.

### PPAT knockout induces HB cell cycle arrest, apoptosis and cell migration impairment

**Fig. 3:**
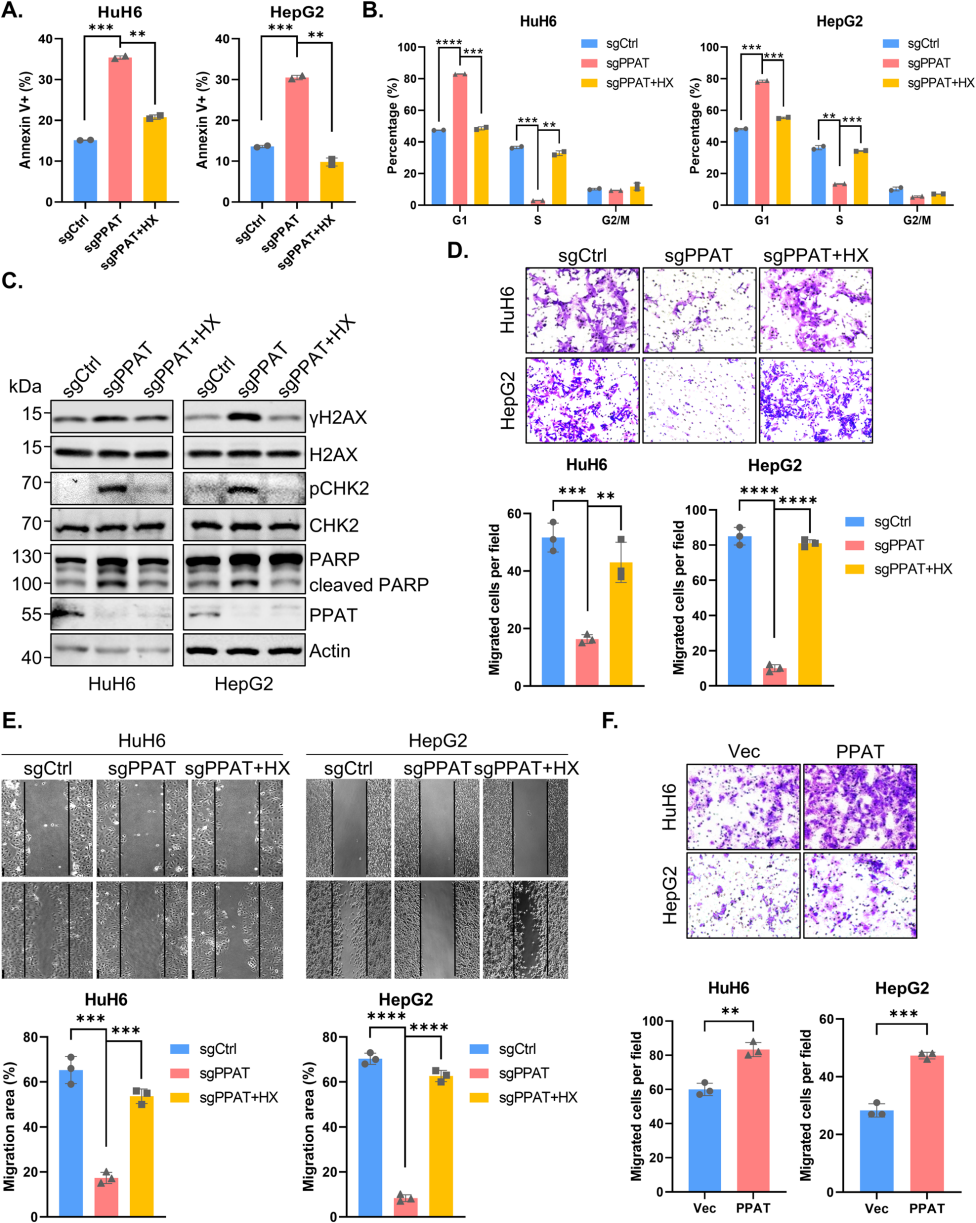
PPAT knockout leads to HB cell cycle arrest, apoptosis and impairment of migration capability. **A** Quantification data of apoptosis analysis of non-targeting control and PPAT knockout HuH6 or HepG2 cells with or without supplementation of 100 μM HX based on flow cytometric analysis (unpaired *t*-test). **B** Quantification data of cell cycle analysis of non-targeting control and PPAT knockout HuH6 or HepG2 cells with or without supplementation of 100 μM HX based on flow cytometric analysis (unpaired *t*-test). **C** Apoptosis and DDR biomarker levels determined by WB analysis. **D** Non-targeting control and PPAT knockout HuH6 or HepG2 cells with or without supplementation of 100 μM HX were subjected to migration assay (upper) and the results were quantified (lower), *n* = 3 (unpaired *t*-test). **E** Non-targeting control and PPAT knockout HuH6 or HepG2 cells with or without supplementation of 100 μM HX were subjected to wound healing assay (upper) and the results were quantified (lower), *n* = 3 (unpaired *t*-test). **F** Vector and PPAT overexpressed HuH6 or HepG2 cells were subjected to migration assay (upper) and the results were quantified (lower), *n* = 3 (unpaired *t*-test).

Impaired cell proliferation can be caused by increased cell cycle arrest or apoptosis. To further investigate the impact of PPAT on HB cells, we conducted cell cycle and apoptosis analyses. Flow cytometry analysis revealed that PPAT knockout significantly augmented the rate of apoptosis in both HuH6 and HepG2 cells, which could be rescued by HX supplementation (Figs. 3A and S3A, C). Additionally, PPAT knockout led to a cell cycle arrest at the G_1_/S phase, a deleterious effect that was readily reversed upon HX supplementation (Figs. 3B and S3B, D). In the latter respect, it is well established that intracellular nucleotide pool insufficiency or imbalance could induce DNA damage response (DDR) [25, 26]. Indeed, we found that the DDR biomarkers γ-H2AX and p-CHK2 (Thr68), as well as the apoptosis marker cleaved PARP, were significantly increased upon PPAT knockout; all these markers returned to baseline levels upon HX supplementation (Fig. 3C). Collectively, our findings suggest that ablation of PPAT leads to a G_1_/S cell cycle arrest, DDR and apoptosis in HB cells.

Metastasis is a crucial process in malignant progression and dissemination and hence a leading cause of HB treatment failure. Therefore, we investigated the impact of PPAT on the migration capability of HB cells. The transwell migration assay revealed that PPAT knockout significantly suppressed the migration of HuH6 and HepG2 cells, whereas HX supplementation restored their migration capability (Figs. 3D and S3E). Similarly, PPAT knockout substantially inhibited wound healing in HuH6 and HepG2 cells, which could be rescued by HX supplementation (Fig. 3E). Furthermore, overexpression of PPAT in HuH6 and HepG2 cells also significantly enhanced cell migration (Fig. 3F). Collectively, these results indicate that PPAT is required for HB cell migration.

### The expression of PPAT is positively correlated with activation of the Wnt/β-catenin signaling pathway

**Fig. 4:**
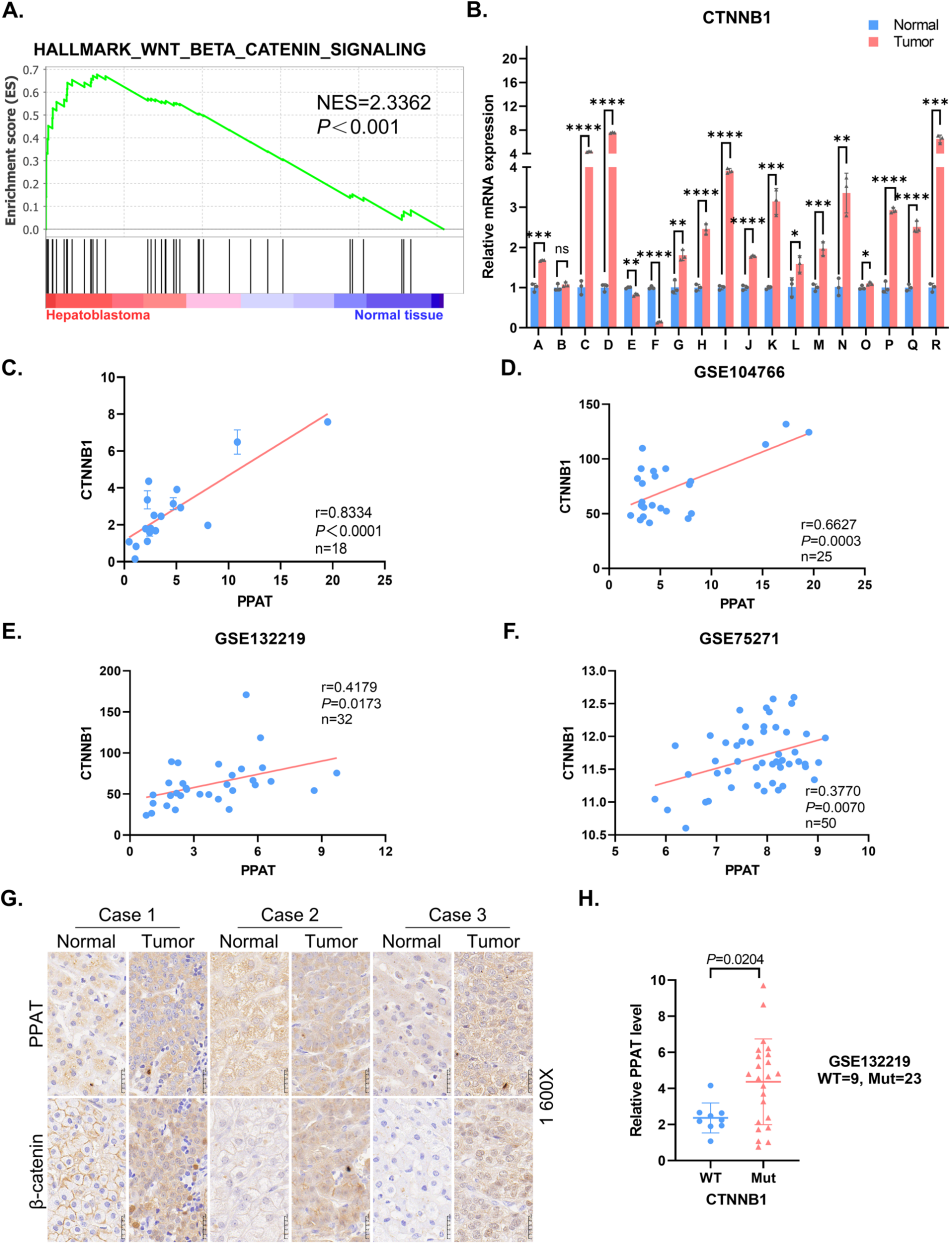
The expression of β-catenin and PPAT is strongly correlated in HB. **A** GSEA evaluating gene signature of Wnt/β-catenin signaling in paired HB and normal tissues (*n* = 10). NES normalized enrichment score. **B** Relative mRNA levels of *CTNNB1* in paired HB and normal tissues (*n* = 18) were determined by RT-qPCR (paired *t*-test). **C** Scatter plots revealed a positive correlation between *PPAT* and *CTNNB1* expression in HB tissues detected by RT-qPCR. The linear best fit lines were shown. The Pearson correlation coefficient (*r*) and *p* values (*p*) were obtained from two-tailed *t*-test. **D**–**F** Scatter plots revealed a positive correlation between *PPAT* and *CTNNB1* expression in HB tissues. Data were from three independent published datasets. The linear best fit lines were shown. The Pearson correlation coefficient (*r*) and *p* values (*p*) were obtained from two-tailed *t*-test. **G** Representative IHC analysis of PPAT and β-catenin in paired HB and normal tissues. **H** The expression of *PPAT* in HB harboring WT and mutant *CTNNB1*. Data were from an independent published dataset (unpaired *t*-test).

Given that HB is driven by Wnt/β-catenin [7, 27], we postulated that the activated Wnt/β-catenin signaling pathway may be implicated in regulation of PPAT expression. GSEA analysis of paired HB sample RNA-seq data verified a significant enrichment of upregulated genes involved in Wnt/β-catenin signaling pathway in HB specimens (Fig. 4A and Table S1). Considering the pivotal role of *CTNNB1*, the gene encoding for β-catenin, in the Wnt/β-catenin signaling pathway and the high prevalence of its activating mutations (50–90%) in HB patients, we consistently found markedly elevated mRNA and protein levels of β-catenin in HB patient samples as compared to their paired normal tissues (Figs. 4B and 1D). Furthermore, we also detected an increased protein level of axis inhibition protein 2 (Axin2), a well-established downstream target of the Wnt/β-catenin signaling pathway, as well as some *CTNNB1* exon deletions in HB samples (Fig. 1D and Table S2). In accord with the clinical specimens, HB cell lines also exhibited significantly higher *CTNNB1* expression (Fig. S4A). Moreover, a robust positive correlation was observed between *PPAT* and *CTNNB1* gene expression in HB specimens (Fig. 4C), which was also supported by the analysis of three independent published datasets (GSE104766, GSE132219 and GSE75271) (Fig. 4D–F). Notably, we also observed a positive correlation between *AXIN2* and *CTNNB1* (Fig. S4B–D). IHC analysis conducted on HB patient specimens further revealed a concordant upregulation of both PPAT and β-catenin expression (Fig. 4G). Meanwhile, the subcellular localization results revealed that β-catenin was predominantly located at cell membrane in the normal tissues, while located at cytoplasm and nucleus in tumor tissues, thereby indicating the Wnt/β-catenin pathway was activated in these HB specimens (Fig. 4G). Remarkably, *PPAT* expression was predominantly increased in patients harboring activating *CTNNB1* mutations (Fig. 4H). Collectively, these findings underscore a positive correlation between *CTNNB1* and *PPAT* expression, indicating the likely regulation of PPAT expression by activated β-catenin.

### β-catenin transcriptionally upregulates *PPAT* expression

**Fig. 5:**
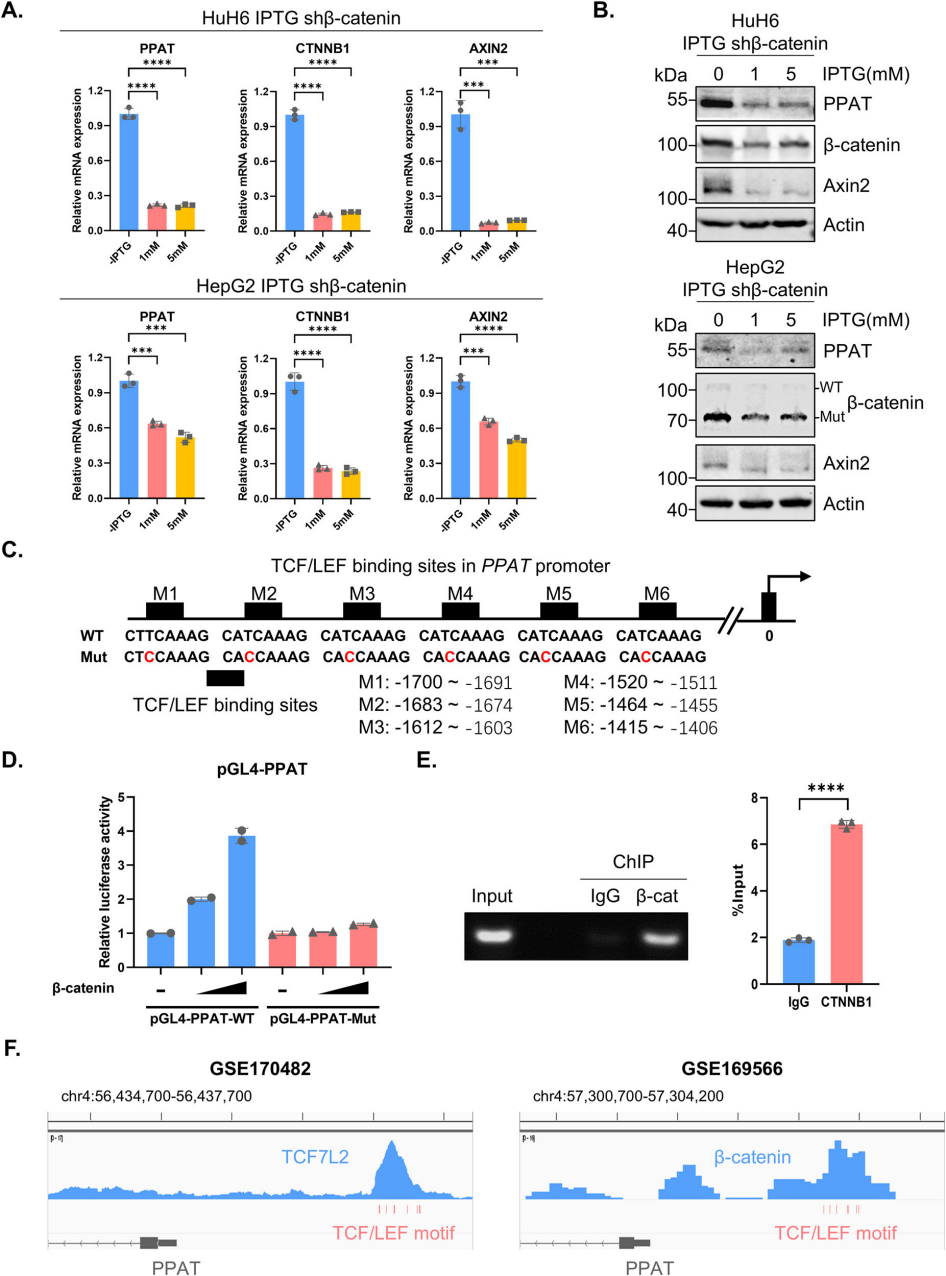
β-catenin transcriptionally activates *PPAT* expression. The relative mRNA (**A**) and protein (**B**) levels of PPAT, β-catenin and Axin2 in inducible β-catenin knockdown cells upon IPTG treatment (1 and 5 mM) for 4 days were determined by RT-qPCR or WB analyses (unpaired *t*-test). **C** The sequences of WT and mutated TCF/LEF binding sites in the *PPAT* promoter region. **D** Luciferase reporter constructs harboring WT and mutated *PPAT* promoter sequence and increasing amounts of β-catenin plasmids were co-transfected into HEK-293T cells, followed by the determination of luciferase activity. **E** β-catenin recruitment to the *PPAT* promoter as determined by ChIP–qPCR (unpaired *t*-test). **F** Snapshots of ChIP-seq data from datasets GSE170482 and GSE169566. The TCF/LEF binding sites in *PPAT* promoter region were highlighted in red below the signal peaks. *PPAT* exon 1 and part of intron 1 were depicted in gray at the bottom, with arrows pointing on them to indicate their antisense direction.

To investigate the correlation between the Wnt/β-catenin signaling pathway and PPAT expression, we used an inducible short hairpin RNA (shRNA) vector to knockdown β-catenin expression in HB cell lines, which was confirmed by RT-qPCR and WB analysis (Fig. 5A, B). Our results showed that depletion of β-catenin led to a decrease in both the mRNA and protein levels of PPAT as well as Axin2 (Figs. 5A, B and S5A, B). In the canonical Wnt/β-catenin signaling pathway, activated Wnt binds to receptors on the plasma membrane to trigger β-catenin activation in the cytoplasm, which then translocates into the nucleus where it interacts with TCF/LEF transcription factors to activate transcription of target genes. Remarkably, we observed that six consensus TCF/LEF binding sites reside in the *PPAT* promoter region (http://jaspar.genereg.net/) (Fig. 5C), suggesting that β-catenin may function as a transcriptional activator of *PPAT* expression. We therefore constructed a pGL4-*PPAT* promoter reporter system and found that β-catenin enhanced the promoter-driven expression of luciferase in a plasmid dose-dependent manner (Fig. 5D). Moreover, mutational inactivation of the consensus binding sites in the *PPAT* promoter markedly blunted β-catenin-mediated luciferase expression (Fig. 5C, D). Furthermore, the WT *PPAT* promoter-driven, as well as the positive control Super 8x TOPFlash promoter-driven expression of luciferase markedly decreased after depletion of β-catenin in HuH6 and HepG2 cells, while the mutant *PPAT* (mutational inactivation of the consensus binding sites) promoter-driven, as well as the negative control Super 8x FOPFlash (TOPFlash mutant) promoter-driven expression of luciferase showed no significant changes when knocking down β-catenin (Fig. S5C). Furthermore, our chromatin immunoprecipitation quantitative PCR (ChIP-qPCR) assay revealed that β-catenin physically binds to the *PPAT* promoter region (Fig. 5E). Of note, we also found a public dataset GSE170482, a TF ChIP-seq on HepG2 targeting TCF7L2 (i.e., TCF4), in ENCODE database and a published dataset GSE169566, a ChIP-seq on HepG2 using β-catenin antibody. We observed significant signal peaks at the putative binding motif area in the promoter region of *PPAT*, which further supported our conclusions (Fig. 5F). Collectively, these findings establish that β-catenin is a bona fide transcriptional activator of *PPAT* expression.

### β-catenin stimulates de novo purine biosynthesis in HB cells

**Fig. 6:**
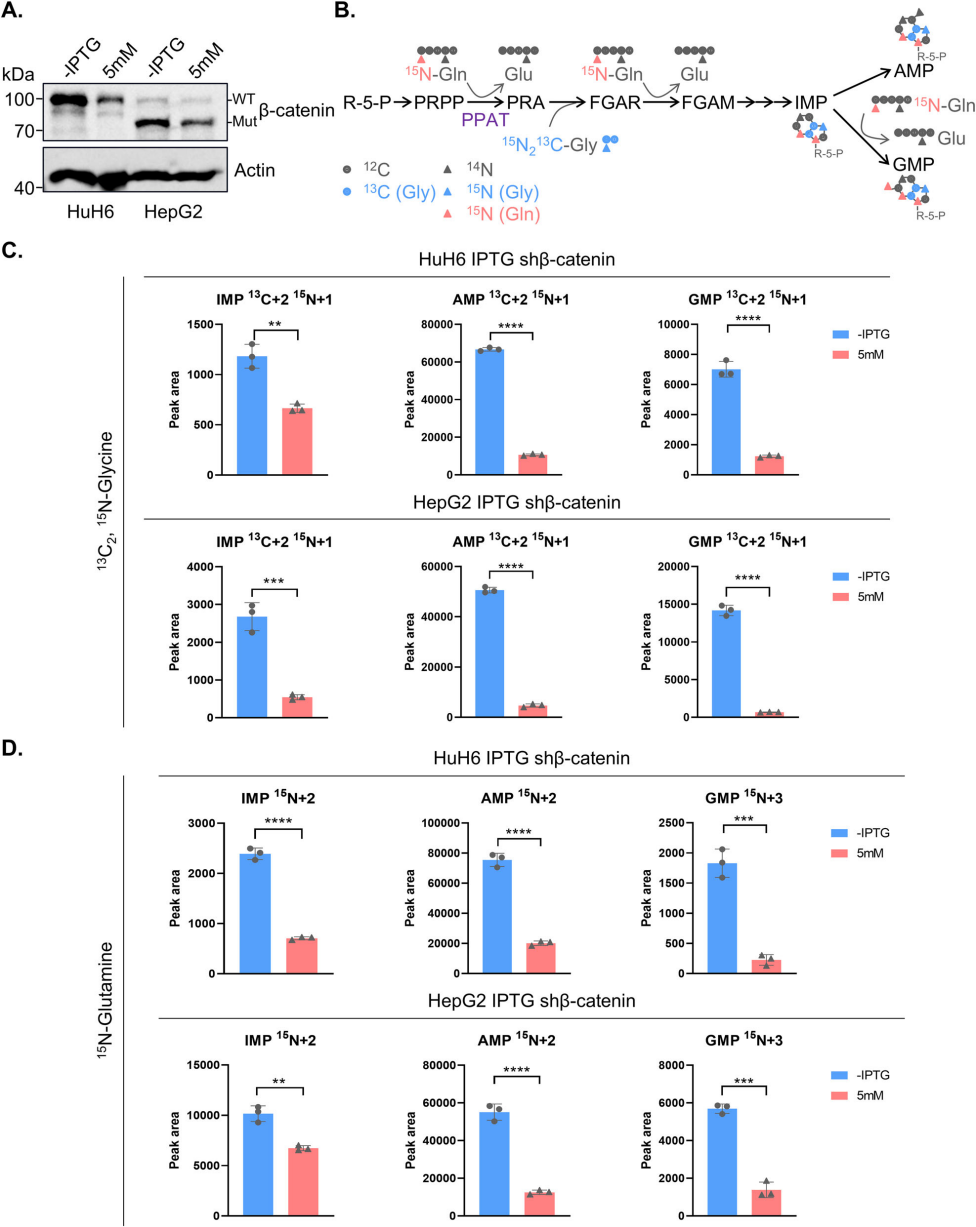
Wnt/β-catenin signaling pathway regulates de novo purine biosynthesis activity. **A** β-catenin protein levels in inducible β-catenin knockdown cells with or without IPTG treatment (5 mM) for 4 days as detected by WB analysis. **B** A summary model for the use of stable isotope labeled glycine and glutamine to trace de novo purine biosynthesis. Relative levels of labeled IMP, AMP and GMP in inducible β-catenin knockdown cells with or without IPTG treatment (5 mM) for 4 days using ^13^C_2_,^15^N-Glycine (**C**) and ^15^N-Glutamine (**D**).

Considering that PPAT serves as the first and rate-limiting enzyme in DNPS, we further explored whether β-catenin could regulate this metabolic pathway in HB. After confirming the effectiveness of β-catenin knockdown (Fig. 6A), we employed stable isotope tracing experiments using labeled glycine (^13^C_2_, ^15^N-glycine) or glutamine (^15^N-glutamine) to assess the activity of DNPS (Fig. 6B). Our liquid chromatography-mass spectrometry (LC-MS) analysis revealed a significant decrease in the de novo biosynthesis of isotope labeled IMP, AMP and GMP by either using ^13^C_2_, ^15^N-glycine or ^15^N-glutamine tracing upon ablation of β-catenin in HuH6 and HepG2 cells (Figs. 6C, D and S5D). These findings demonstrate that depletion of β-catenin led to a suppression of PPAT expression, thereby markedly disrupting DNPS in HB cells.

### Therapeutic strategy targeting de novo purine biosynthesis in HB with β-catenin mutations

**Fig. 7:**
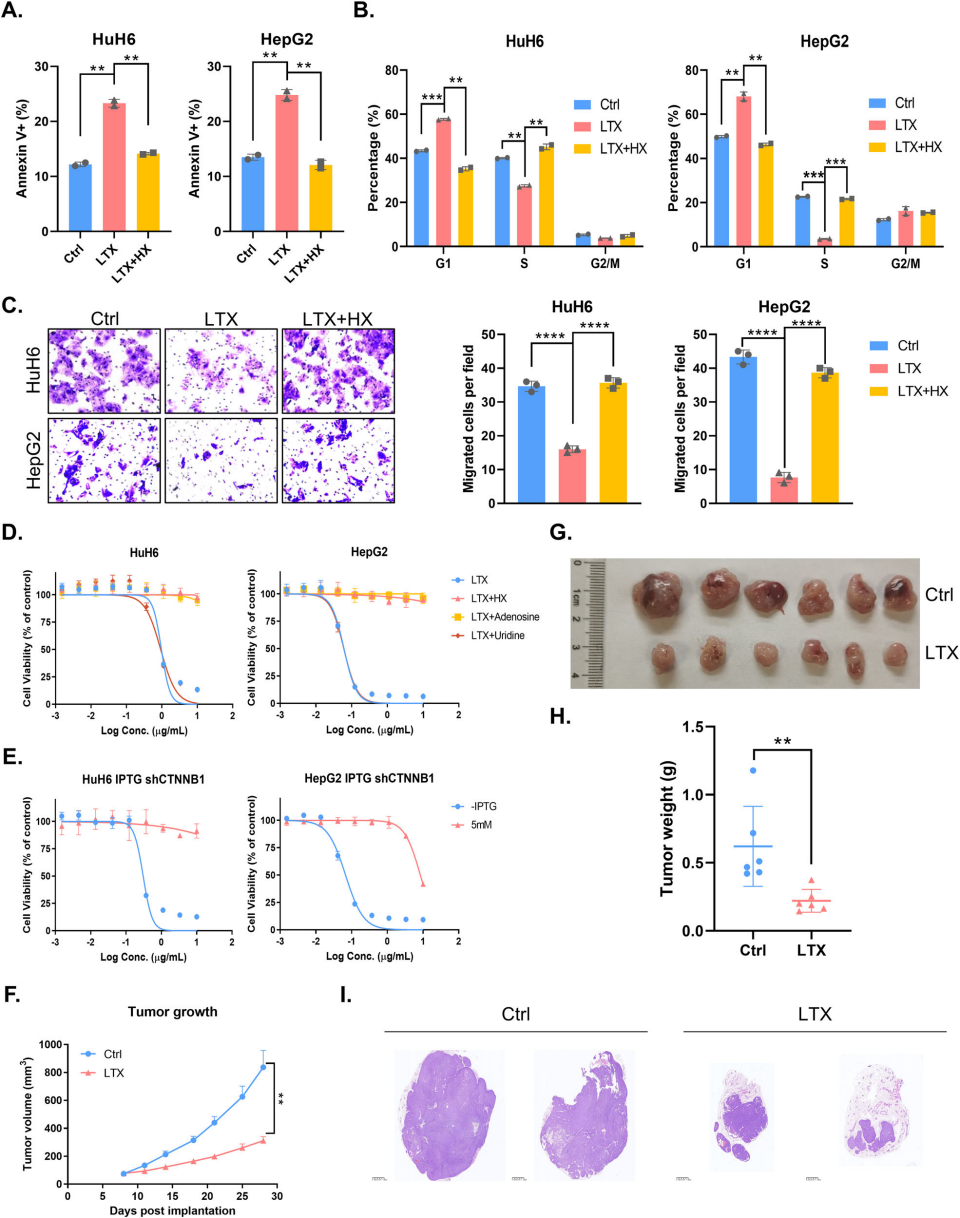
Targeting de novo purine biosynthesis by the GART inhibitor lometrexol inhibits β-catenin mutant HB tumor growth. Apoptosis (**A**) and cell cycle analysis (**B**) of HuH6 or HepG2 cells upon treatment with 30 μM LTX with or without 100 μM HX as determined by flow cytometric analysis (unpaired *t*-test). **C** Migration assay of HuH6 or HepG2 cells upon 30 μM LTX treatment with or without 100 μM HX and quantification analysis (unpaired *t*-test). **D** Cell viability of HuH6 or HepG2 cells upon LTX treatment (10 µg/ml) with different nucleosides at 72 h. **E** Cell viability of HuH6 or HepG2 cells with or without β-catenin knockdown in response to LTX treatment (10 µg/ml). **F** LTX (40 mg/kg) effectively suppressed HB tumor growth in nude mice subcutaneously implanted with indicated HuH6 cells. Tumor volume was monitored twice a week (unpaired *t*-test). Tumor size (**G**) and weight (**H**) at the end point (unpaired *t*-test) of the in vivo experiment. **I** H&E staining of tumor tissues depicted in (**F**).

The aforementioned results provide compelling evidence that β-catenin upregulates DNPS via enhancement of PPAT expression, thereby promoting HB progression and metastasis. Consequently, we hypothesized that drug targeting of DNPS could serve as an efficacious therapeutic strategy to inhibit HB progression. Lometrexol (LTX) specifically targets and blocks GART activity, thereby abolishing DNPS. Indeed, LTX significantly induced apoptosis in HB cells in vitro, which could be effectively rescued by HX supplementation (Fig. 7A). Moreover, LTX treatment induced a cell cycle arrest at the G_1_/S phase in HB cells, while restoration of cell cycle progression occurred upon HX supplementation (Fig. 7B). Remarkably, inhibition of cell migration caused by LTX was also reversed by HX supplementation (Fig. 7C). Of note, our drug sensitivity assay indicated that cytotoxicity of LTX towards HB cells could only be counteracted by HX and adenosine supplementation, but not by supplementation with the pyrimidine uridine (Fig. 7D). Finally, we also conducted drug sensitivity assay upon LTX treatment in β-catenin knockdown HB cells. Our results showed that HB cells acquired resistance to LTX when β-catenin was knockdown, thereby indicating that LTX is effective in HB suppression in β-catenin-activated tumors (Fig. 7E). These findings demonstrate that LTX blocks DNPS, thereby suppressing HB cell proliferation in vitro.

We therefore validated these findings in vivo utilizing an HB xenograft mouse model. Our results showed that LTX significantly blocked HB growth in vivo which corroborated the in vitro findings (Figs. 7F–H and S6A). Hematoxylin and Eosin (H&E) staining also revealed the reduced cell proliferation in HB xenografts following LTX administration (Fig. 7I). To further distinguish between the residual tumor tissue and the non-tumor tissue after LTX treatment, we used Perilipin-1 IHC staining. Perilipin-1 is a surface protein of lipid droplets in adipocytes. Indeed, large Perilipin-1-positive non-tumor areas were apparent in LTX treated tumors, whereas the the non-treated control tumors were dominated by tumor tissue with minimal Perilipin-1 positive non-tumor areas (Fig. S6B). This finding further established the potent anticancer activity of LTX. Collectively, these results highlight the therapeutic potential of targeting DNPS by LTX as a treatment strategy for activated mutant β-catenin-driven HB.

## Discussion

The current study revealed a key role for activated mutant β-catenin signaling in the enhancement of DNPS via upregulation of PPAT expression, highlighting purine biosynthesis as a key anabolic program in β-catenin-dependent HB cell proliferation and migration both in vitro and in vivo. Tumor cells often display enhanced DNPS due to their rapid cell proliferation and hence maintain the increased intracellular pool of nucleotides for RNA synthesis and DNA replication [12, 28]. Thus, multiple key enzymes in the DNPS pathway are upregulated, constituting promising therapeutic targets in various cancers [23, 24, 29, 30]. Consistently, we herein discovered that DNPS, along with its rate-limiting enzyme PPAT, were aberrantly upregulated in HB tumors. Pharmacological targeting or genetic repressing of the enhanced DNPS markedly blocked HB cell proliferation, colony formation and migration. These also induced cell cycle arrest at the G_1_/S phase, culminating in apoptosis. The suppressive impact of PPAT silencing on HB cells in vitro was readily reproducible in vivo using an HB xenograft mouse model. We consequently found an enhanced β-catenin-mediated *PPAT* transcriptional upregulation which markedly boosted DNPS, thereby forming a specific metabolic vulnerability that could be efficiently targeted by specific enzyme inhibitors of the DNPS pathway. Our findings highlight the paramount importance of target DNPS inhibition in activated mutant β-catenin-driven HB and possibly in other cancers as well [13, 31–33].

Multiple factors have been reported to regulate DNPS [17, 22, 34]: (1) The key proto-oncogene *MYCN* was found to transcriptionally upregulate PAICS and enhance DNPS, thereby promoting neuroblastoma progression [29]. (2) The central tumor suppressor p53 was shown to transcriptionally repress inosine monophosphate dehydrogenase (IMPDH), guanine monophosphate synthase (GMPS) and methylenetetrahydrofolate dehydrogenase 2 (MTHFD2), which are directly or indirectly involved in DNPS [35–38]. p53 also inhibited ATIC, another DNPS enzyme, via its acetylation by lysine acetyltransferase 2B (KAT2B); moreover, p53-deficient cells were found to be more sensitive than wild type (WT) p53 cells to ATIC inhibition [39]. (3) The RAS/RAF-ERK signaling pathway stimulated DNPS via direct phosphorylation of PFAS at Thr619 by extracellular regulated protein kinases 2 (ERK2), which is required for tumor growth [40]. Consistently, six enzymes in DNPS were found to undergo as many as 7 types of post-translational modifications including methylation, acetylation, phosphorylation and ubiquitination; these also included RAC-α serine/threonine-protein kinase (AKT)-mediated PPAT phosphorylation at Thr397 [41]. Clearly however, further in-depth studies are warranted to pin-point the functional consequences of these multiple post-translational modifications of enzymes in the DNPS pathway. (4) p16/CDKN2A is a tumor suppressor encoded by *CDKN2A*, the expression of which is lost in ~50% of human cancers [42, 43]. It was recently reported that several DNPS genes are essential for the survival of melanoma cells which lost p16/CDKN2A [33]. These tumor cells with low p16/CDKN2A expression exhibited sensitivity to various DNPS inhibitors, including LTX (GART inhibitor), mycophenolic acid, VX-944 (IMPDH inhibitor) and 6-mercaptopurine (a purine analog), as well as the antifolates methotrexate and aminopterin (dihydrofolate reductase [DHFR] inhibitors). Previous studies have established a group of murine HB models driven by patient-derived β-catenin mutations or other mutations such as YAP and NFE2L2 [44, 45]. We did not observe up-regulated *Ppat* in a variety of murine HB tumor samples due to the lack of consensus TCF/LEF binding site in the mouse *Ppat* promoter region (data not shown). It is possible that future studies may identify factors that could regulate the expression/activity of PPAT or DNPS in HB or other tumors.

Six enzymes in the DNPS pathway including PPAT, GART, PFAS, PAICS, ADSL and ATIC have been previously shown to form a multi-enzyme complex known as the purinosome, which facilitates the purine metabolic flux response in rapidly dividing cells [46]. The assembly/disassembly of the purinosome can be dynamically influenced by cell cycle progression, intracellular purine nucleotide levels, mTORC1, G-protein-coupled receptors (GPCRs), heat shock protein 90 (Hsp90) and heat shock protein 70 (Hsp70) [47–50]. In this respect, we herein found that the six enzymes involved in purinosome formation were uniformly upregulated in HB. However, apart from PPAT, the underlying mechanisms upregulating the rest five enzymes remain unclear and hence deserve further investigation. Whether Wnt/β-catenin hyperactivation could enhance purinosome formation in HB remains to be determined.

Increasing evidence indicates that cancer cells heavily rely on DNPS when compared to normal cells which are dependent on the salvage pathway [23, 24]. For example, acute myeloid leukemia (AML) cells were found to be highly dependent on PAICS as predicted from DepMap datasets and confirmed by CRISPR-Cas9 screening. Moreover, PAICS inhibition showed little toxicity to normal hematopoiesis but was particularly cytotoxic to AML cells [24]. Colorectal cancer cells with MLL3/4-COMPASS mutations increased DNPS flux and were selectively sensitive to LTX treatment [17]. Consistently, our findings demonstrated that HB cells with activated mutant β-catenin exhibited an enhanced dependence on PPAT (i.e., DNPS) rather than on HPRT1 (i.e., salvage) to sustain cell proliferation, indicating that targeted inhibition of DNPS could be an effective therapeutic strategy to halt rapidly proliferating HB cells. Indeed, our current study suggested that pharmacological targeting of enzymes in the DNPS pathway like LTX-mediated inhibition of GART readily block HB progression in vitro and in vivo. Furthermore, blocking one-carbon metabolism and the folate cycle using potent antifolates like methotrexate and pralatrexate, both of which target the key enzyme DHFR [13], are likely to achieve a synergistic antitumor activity when combined with LTX. The rationale behind this combination is that blocking the formation of tetrahydrofolate cofactors, which serve as one-carbon donors in DNPS, should synergize with direct DNPS enzyme inhibitors like LTX. Along this vein, combination of LTX with IMPDH inhibitors like mycophenolic acid and VX-944 could also prove a powerful synergistic combination. As monotherapy of various human cancers is typically insufficient to achieve tumor eradication, experimental exploration of the above rationale-based drug combinations is warranted.

Our findings revealed that enhanced DNPS is a bona fide metabolic vulnerability driven by activated mutant β-catenin in HB. Notably, the rate-limiting DNPS enzyme PPAT was markedly upregulated and was found essential for HB tumor cell proliferation, migration and progression, thereby displaying a pro-tumorigenic role in HB. The hyperactive mutant β-catenin transcriptionally stimulated *PPAT* expression, thereby enhancing DNPS flux to promote HB tumor progression, which could be efficiently targeted by the specific DNPS inhibitor LTX. It is plausible that pharmacological inhibition of some DNPS enzymes may bear broad therapeutic implications for various cancers which are driven by an activated Wnt/β-catenin pathway or alternatively display enhanced DNPS activity driven by other factors.

## Supplementary information

Supplemental figures

Supplemental tables

Original western blots

## Data Availability

The RNA-seq data generated in this study have been deposited to Open Archive for Miscellaneous Data (OMIX) database of China National Center for Bioinformation (CNCB), https://ngdc.cncb.ac.cn/omix/ (Accession No. OMIX004146). The other data and materials used in the current study are available from the corresponding authors upon reasonable request.
