# Supplementary material for: Therapeutic targeting de novo purine biosynthesis driven by β-catenin-dependent PPAT upregulation in hepatoblastoma
Source: Cell Death Dis. 2025 Mar 17;16(1):179. doi: 10.1038/s41419-025-07502-6 (PMC11914223; doi:10.1038/s41419-025-07502-6)
Supplement: Supplementary file 1 — Supplemental figures [file 41419_2025_7502_MOESM1_ESM.pdf]

Figure S1. The *de novo* purine biosynthesis pathway is upregulated in HB and is associated with poor prognosis.

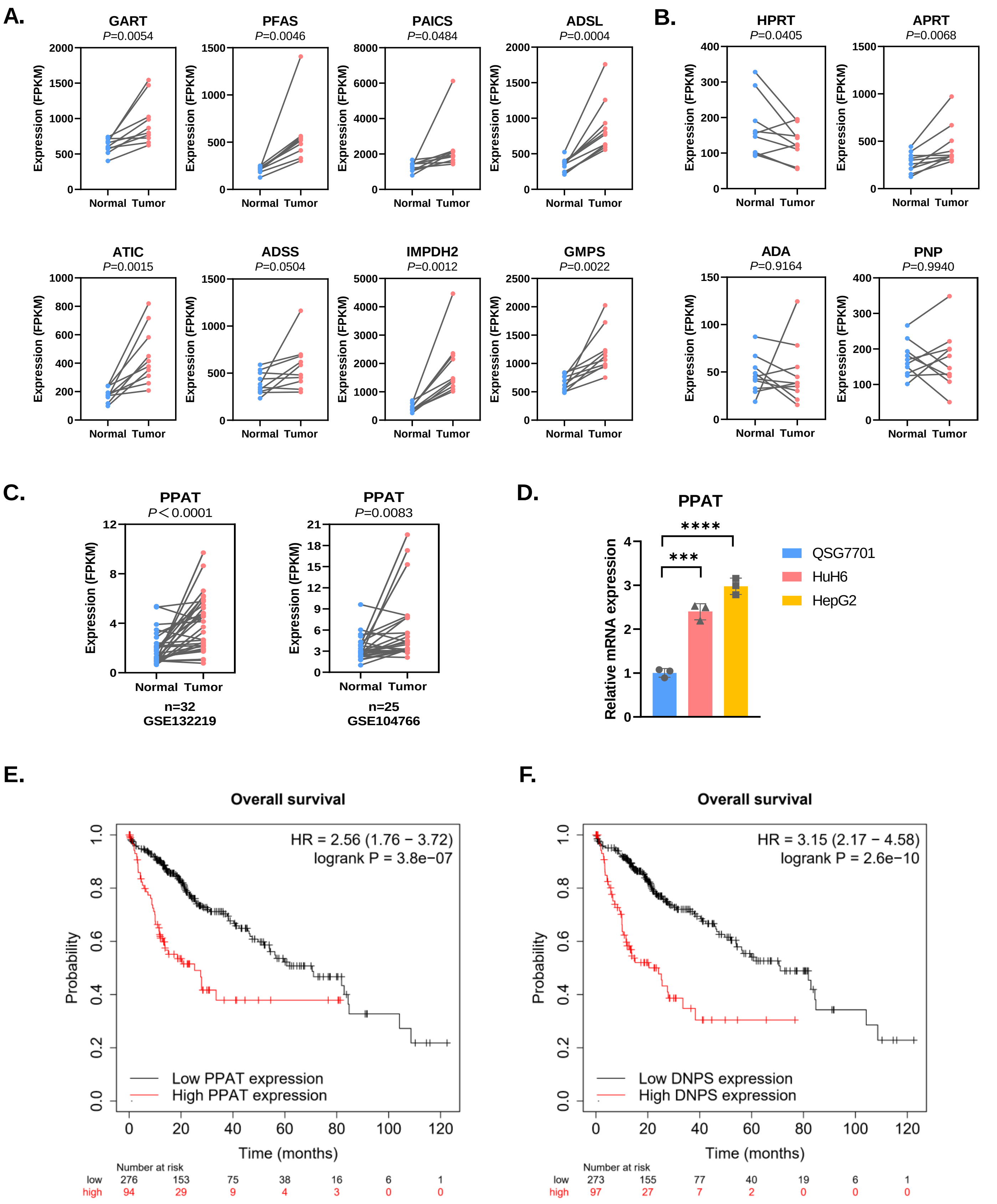

Legend on next page

Figure S1. The *de novo* purine biosynthesis pathway is upregulated in HB and is associated with poor prognosis.

Figure S1. The *de novo* purine biosynthesis pathway is upregulated in HB and is associated with poor prognosis.

(A) mRNA levels of genes involved in the *de novo* purine biosynthesis pathway in 10 pairs of HB and normal tissue as determined by RNA-Seq.

(B) RNA levels of genes involved in salvage purine biosynthesis pathway in 10 pairs of HB and normal tissues as determined by RNA-Seq.

(C) mRNA levels of PPAT in two independent public datasets.

(D) Relative mRNA levels of PPAT in normal liver cells (QSG7701) and HB cells (HuH6 and HepG2) determined by RT-qPCR (unpaired t-test).

(E) Kaplan-Meier plots for overall survival in public liver cancer datasets. Red, high expression of PPAT; black, low expression of PPAT; HR, hazard ratio.

(F) Kaplan-Meier plots for overall survival in public liver cancer datasets. Red, high expression of 6 DNPS genes (PPAT, GART, PFAS, PAICS, ADSL and ATIC); black, low expression of 6 DNPS genes; HR, hazard ratio.

Figure S2. PPAT is essential for HB cell proliferation.

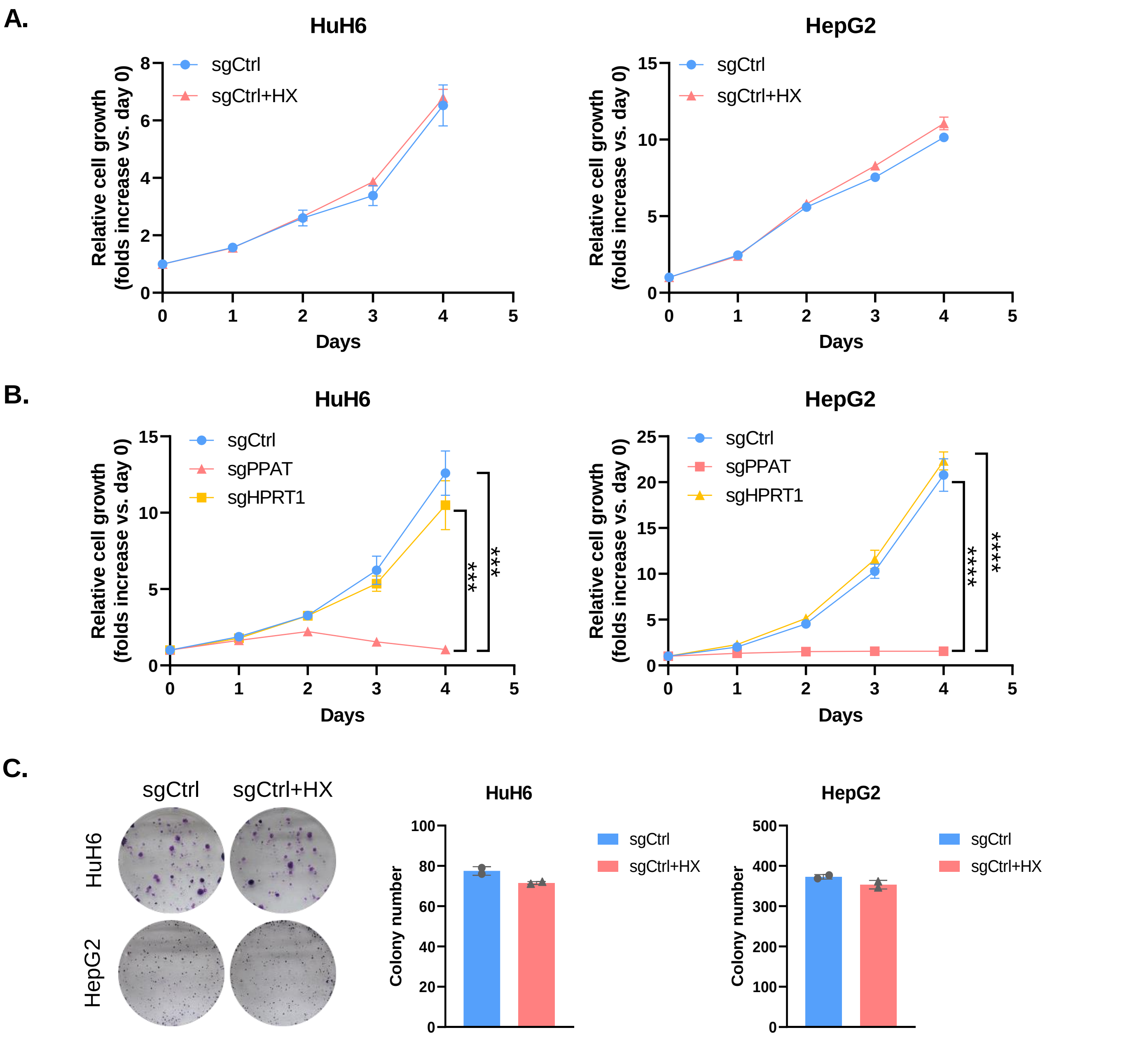

Figure S2. PPAT is essential for HB cell proliferation.

(A) Growth curves were generated for non-targeting control knockout HuH6 or HepG2 cells with or without supplementation of 100 μM HX. Each point was assayed in hexaplicates.

(B) Growth curves were generated for non-targeting control, PPAT knockout and HPRT1 knockout HuH6 or HepG2 cells. Each point was assayed in triplicates (unpaired t test).

(C) Non-targeting control knockout HuH6 or HepG2 cells with or without supplementation of 100 μM HX, were subjected to colony formation assay (left), quantification data (right).

Figure S3. PPAT knockout leads to HB cell cycle arrest, apoptosis and impairment of migration capability.

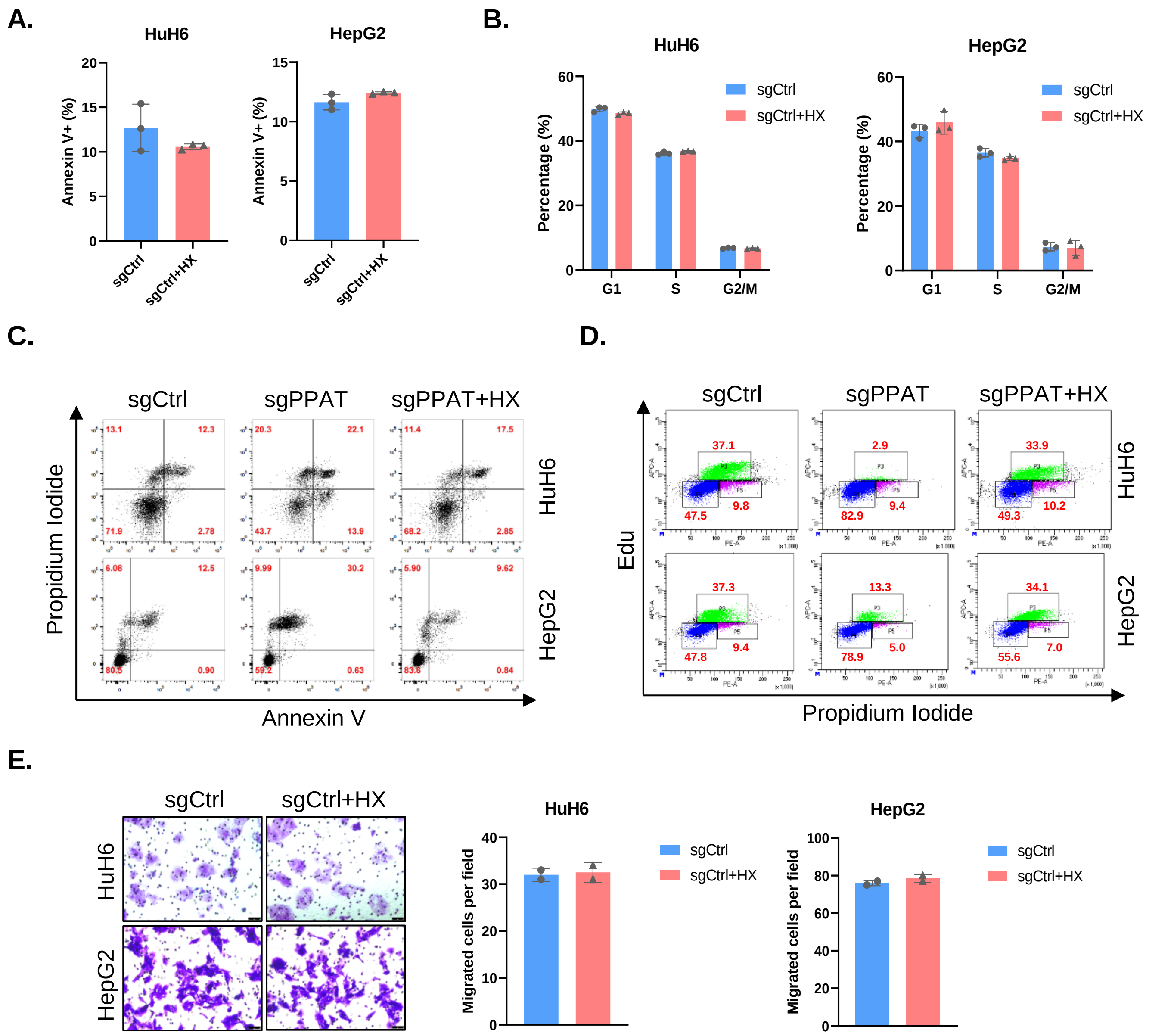

Figure S3. PPAT knockout leads to HB cell cycle arrest, apoptosis and impairment of migration capability.

(A-B) Quantification data of apoptosis (A) and cell cycle (B) analysis of non-targeting control knockout HuH6 or HepG2 cells with or without supplementation of 100 µM HX based on flow cytometric analysis.

(C-D) Representative image of apoptosis (D) and cell cycle (E) analysis of non-targeting control and PPAT knockout HuH6 or HepG2 cells with or without supplementation of 100 µM HX based on flow cytometric analysis.

(E) Non-targeting control knockout HuH6 or HepG2 cells with or without supplementation of 100 µM HX were subjected to migration assay (left) and the results were quantified (right).

Figure S4. The expression of β-catenin and PPAT is strongly correlated in HB.

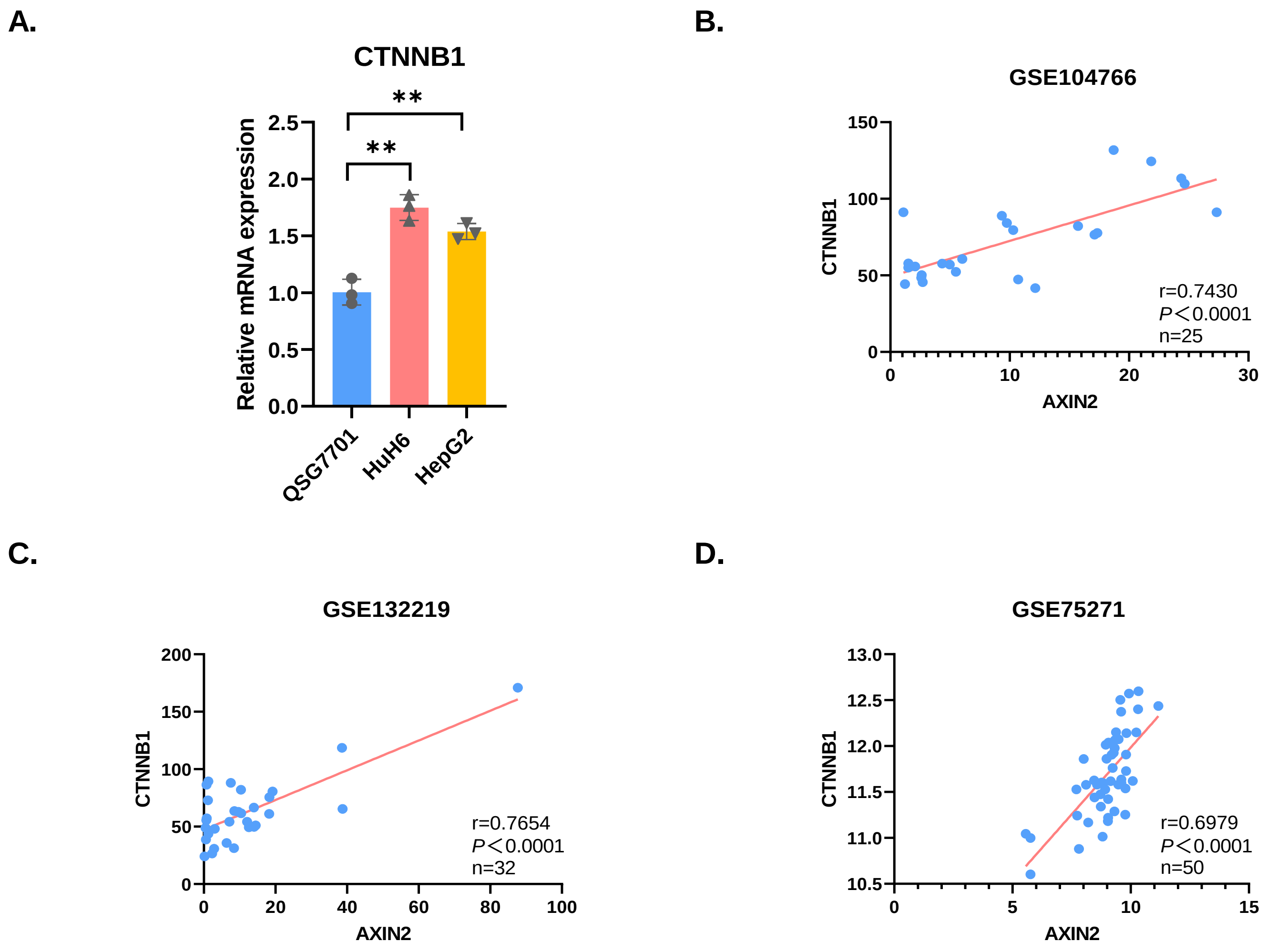

Figure S4. The expression of β-catenin and PPAT is strongly correlated in HB.

(A) Relative mRNA levels of *CTNNB1* in normal liver cells (QSG7701) and HB cells (HuH6 and HepG2) were determined by RT-qPCR (unpaired t-test).

(B-D) Scatter plots revealed a positive correlation between *AXIN2* and *CTNNB1* expression in HB tissues. Data were from three independent published datasets. The linear best fit lines were shown. The Pearson correlation coefficient (r) and p-values (P) were obtained from two-tailed t-test.

Figure S5. β-catenin transcriptionally activates PPAT expression and regulates *de novo* purine biosynthesis activity.

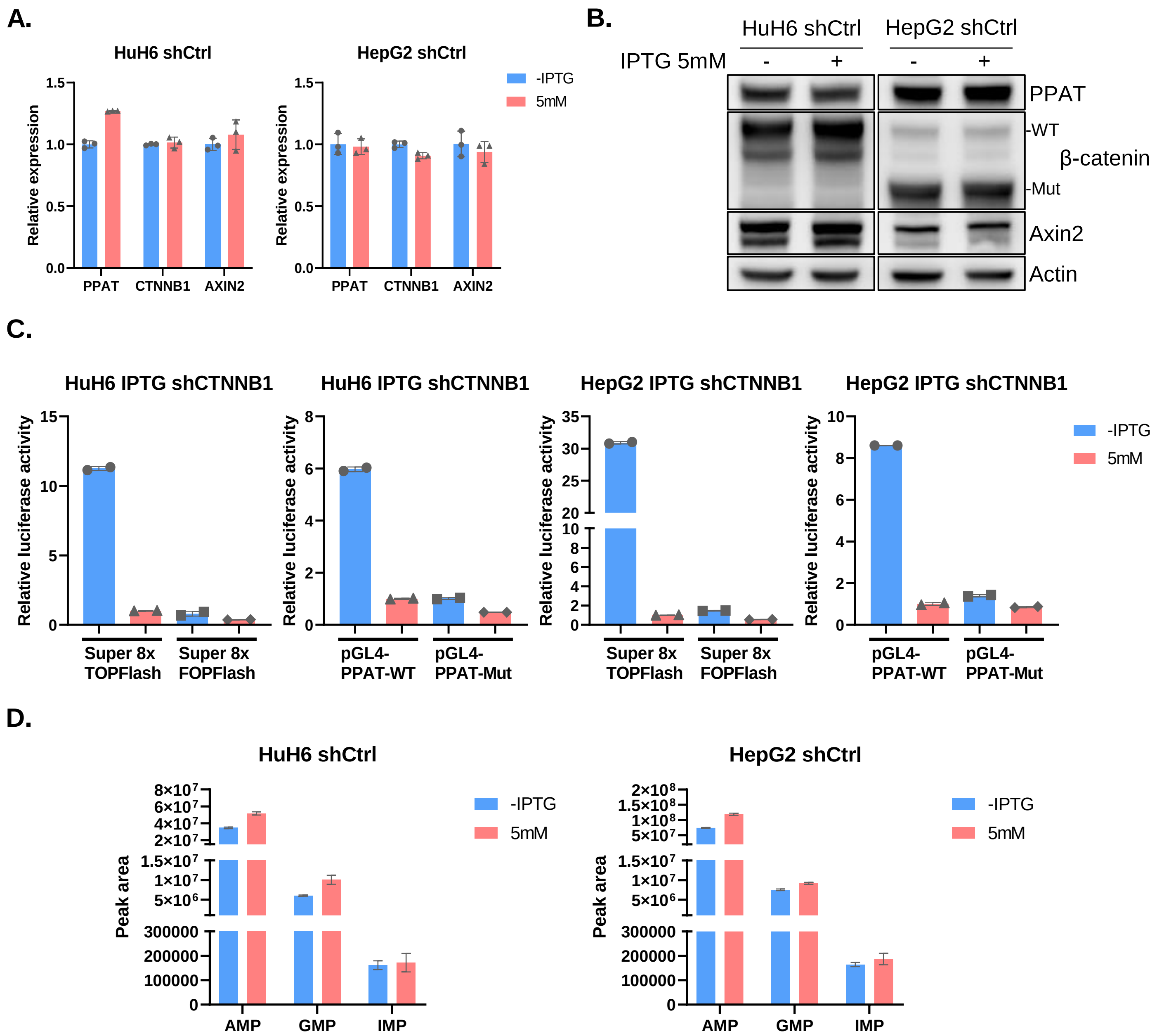

Figure S5. β-catenin transcriptionally activates PPAT expression and regulates *de novo* purine biosynthesis activity.

(A-B) The relative mRNA (A) and protein (B) levels of PPAT, β-catenin and Axin2 in inducible non-targeting control knockdown cells with or without IPTG treatment (5 mM) for 4 days were determined by RT-qPCR or WB analyses.

(C) Either Super 8x TOPFlash and FOPFlash (TOPFlash mutant) or luciferase reporter constructs harboring WT and mutant *PPAT* promoter sequence were transfected into inducible β-catenin knockdown HuH6 or HepG2 cells with or without IPTG treatment (5mM) for 4 days, followed by the determination of luciferase activity.

(D) Relative levels of AMP, GMP and IMP in inducible non-targeting control knockdown cells with or without IPTG treatment (5mM) for 4 days were determined by LC-MS

Figure S6. LTX inhibits β-catenin mutant HB tumor growth.

A.

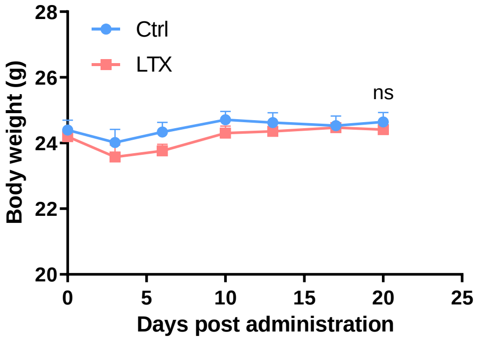

B.

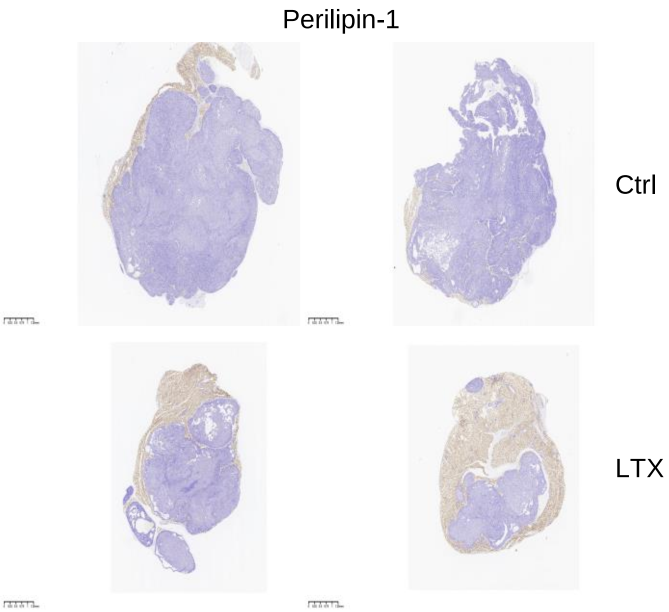

Figure S6. LTX inhibits β-catenin mutant HB tumor growth.

(A) The body weight at the indicated time of the *in vivo* LTX treatment (40 mg/kg) experiment (unpaired t-test).

(B) Perilipin-1 staining of tumor tissues depicted in figure 7G.
