# Supplementary material for: Therapeutic targeting de novo purine biosynthesis driven by β-catenin-dependent PPAT upregulation in hepatoblastoma
Source: Cell Death Dis. 2025 Mar 17;16(1):179. doi: 10.1038/s41419-025-07502-6 (PMC11914223; doi:10.1038/s41419-025-07502-6)
Supplement: Supplementary file 2 — Supplemental tables [file 41419_2025_7502_MOESM2_ESM.pdf]

Supplemental Table 1. The main clinical and pathological features of 10 HB patients involved in GSEA analysis.

Patient ID	Sex	Age at diagnosis (month)	Histology	PRETEXT	Tumor size (cm^3)	AFP at diagnosis (ng/ml)	Metastasis	AFP at final test	Events （death=0, survival=1)	CTNNB1 mutation
HB17	M	5	NA	III	444.96	12600	NO	197.58	1	p.A5_A80 del
HB20	M	1	MIX	II	432	47528	NO	/	0	p.Y30_D32 del
HB21	M	4	MIX	/	/	52.98	NO	/	0	WT
HB23	M	57	NA	IV	/	>120000	YES (Lung)	>120000	0	WT
HB24	M	39	E	/	480	3064	NO	348.63	1	WT
HB25	F	23	MIX	III	240	>120000	NO	365.7	1	p.T41A
HB26	F	27	NA	III	84	109371	NO	726.53	1	WT
HB27	F	4	NA	IV	/	1036.51	NO	210.14	1	p.G34V, p.R469C
HB28	F	6	MIX	III	/	>120000	NO	4835.44	1	p.A5_A80 del
HB29	F	17	NA	III	210	2971.66	NO	2674.5	1	p.A5_A80 del

M: Male; F: Female; E: Epithelial; MIX: Mixed epithelial and mesenchymal; NA: Unknown

Supplemental Table 2. The CTNNB1 mutation status of 8 HB patients involved in WB analysis.

Sample ID	CTNNB1 mutation
I	p.S29F, p.S33F
II	WT
III	WT
IV	p.W25_D32 del
V	p.S23_S33 del
VI	p.Y30_A80 del
VII	p.W25_A80 del
VIII	p.W25_I140 del
