# Supplementary figures and images for: Therapeutic targeting de novo purine biosynthesis driven by β-catenin-dependent PPAT upregulation in hepatoblastoma
Source: Cell Death Dis. 2025 Mar 17;16(1):179. doi: 10.1038/s41419-025-07502-6 (PMC11914223; doi:10.1038/s41419-025-07502-6)

Figure 2A

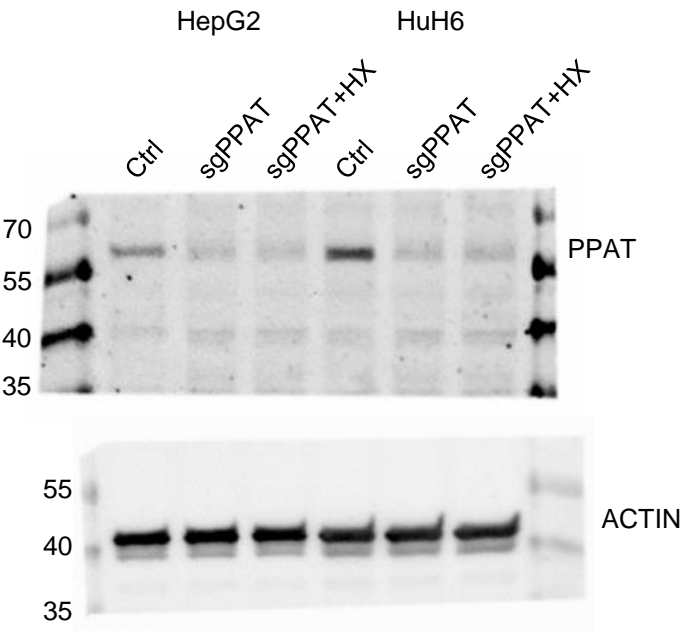

Figure 1D

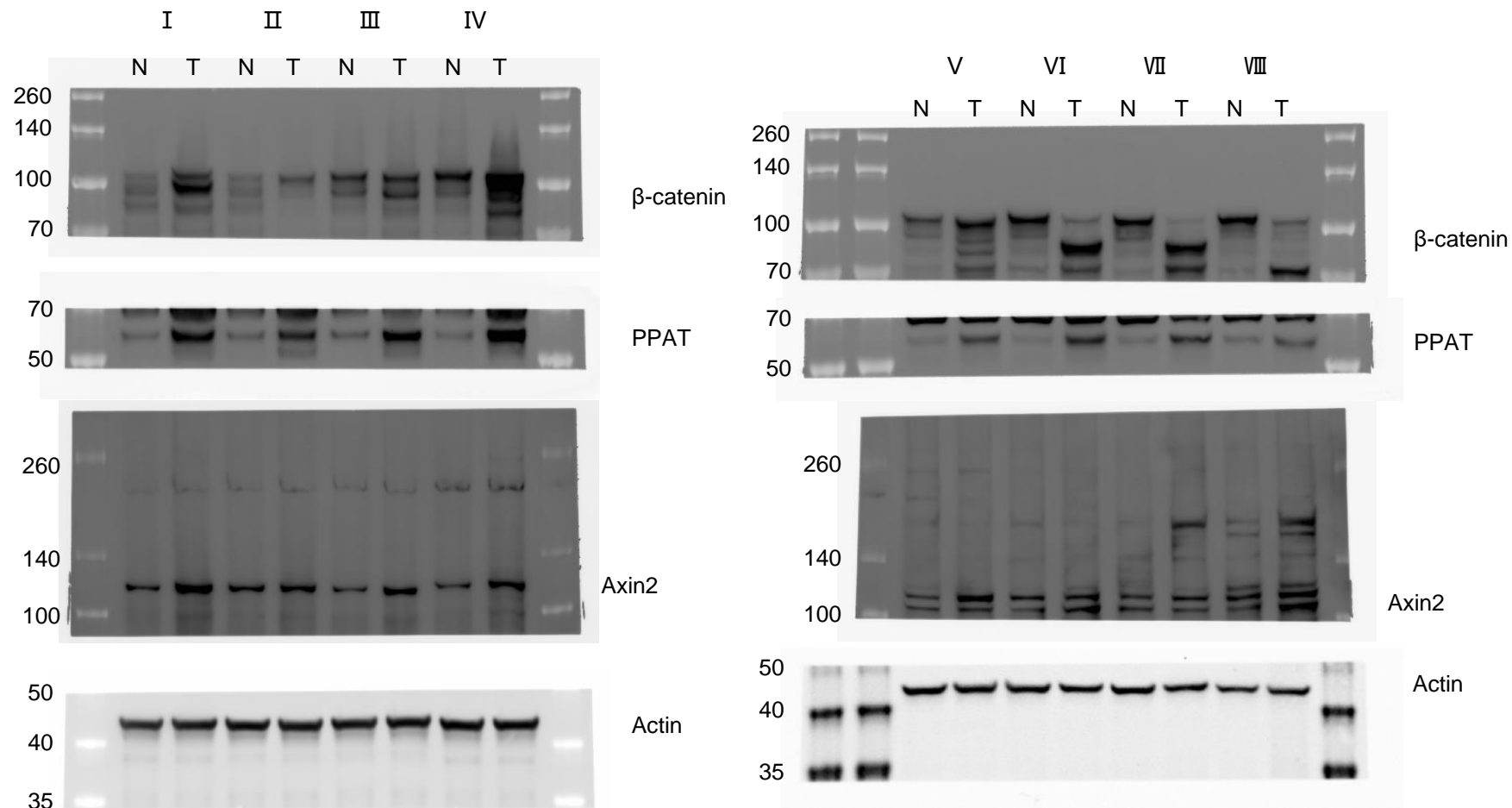

Figure 3C

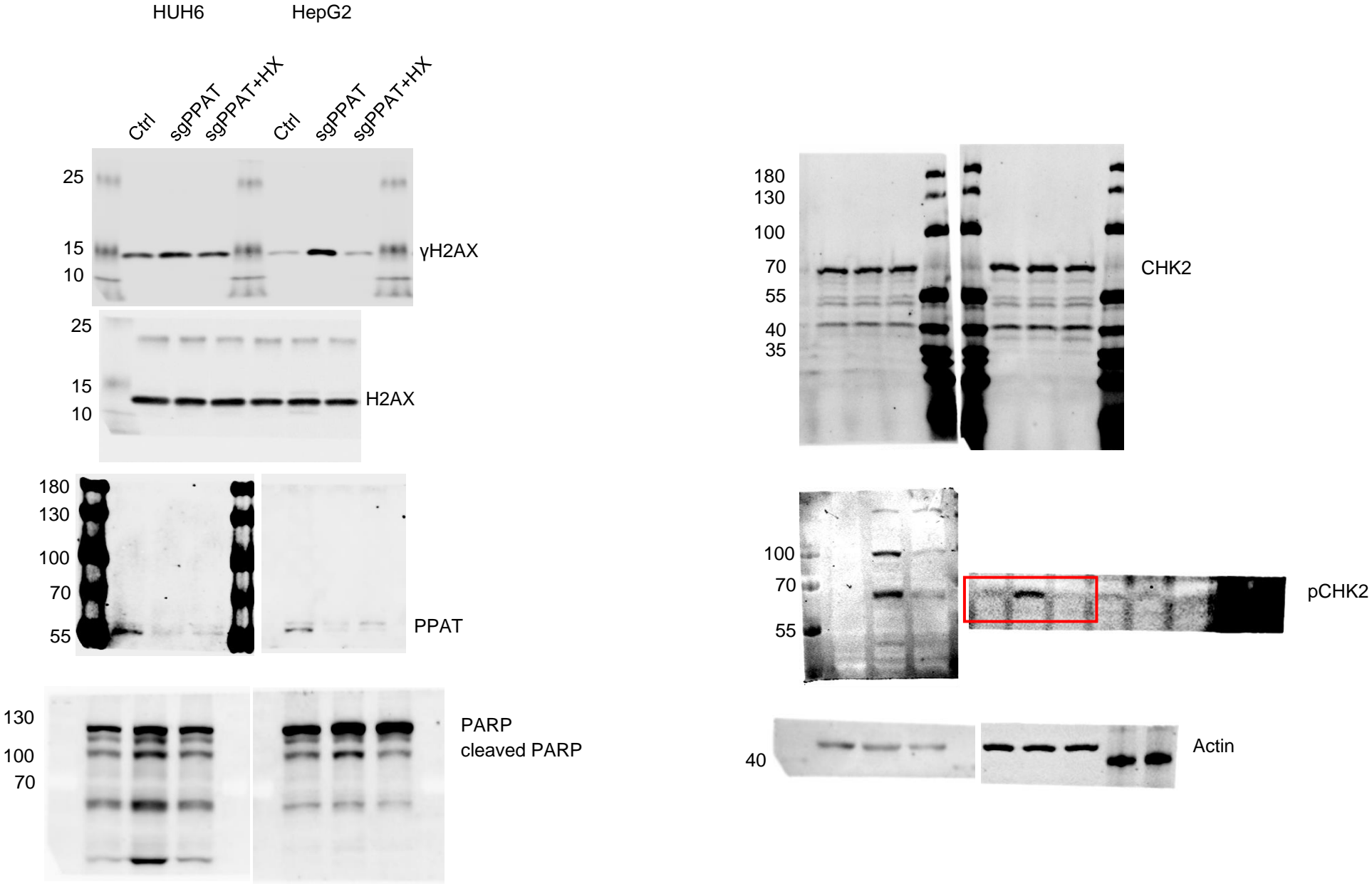

Figure 5B

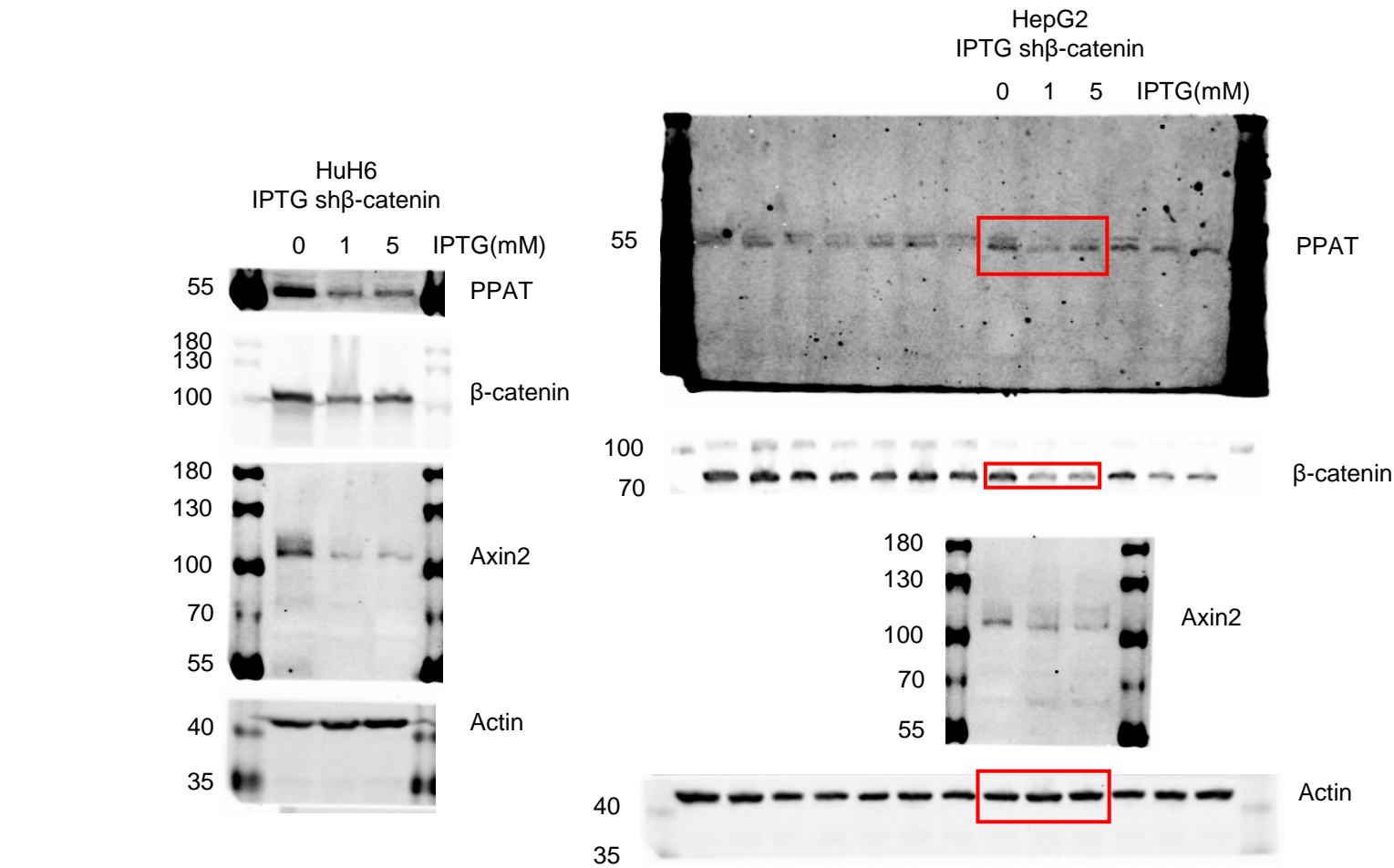

Figure 6A

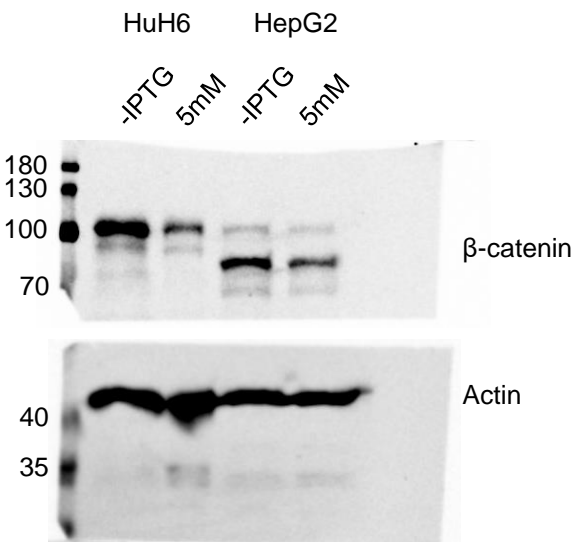

Figure S5B

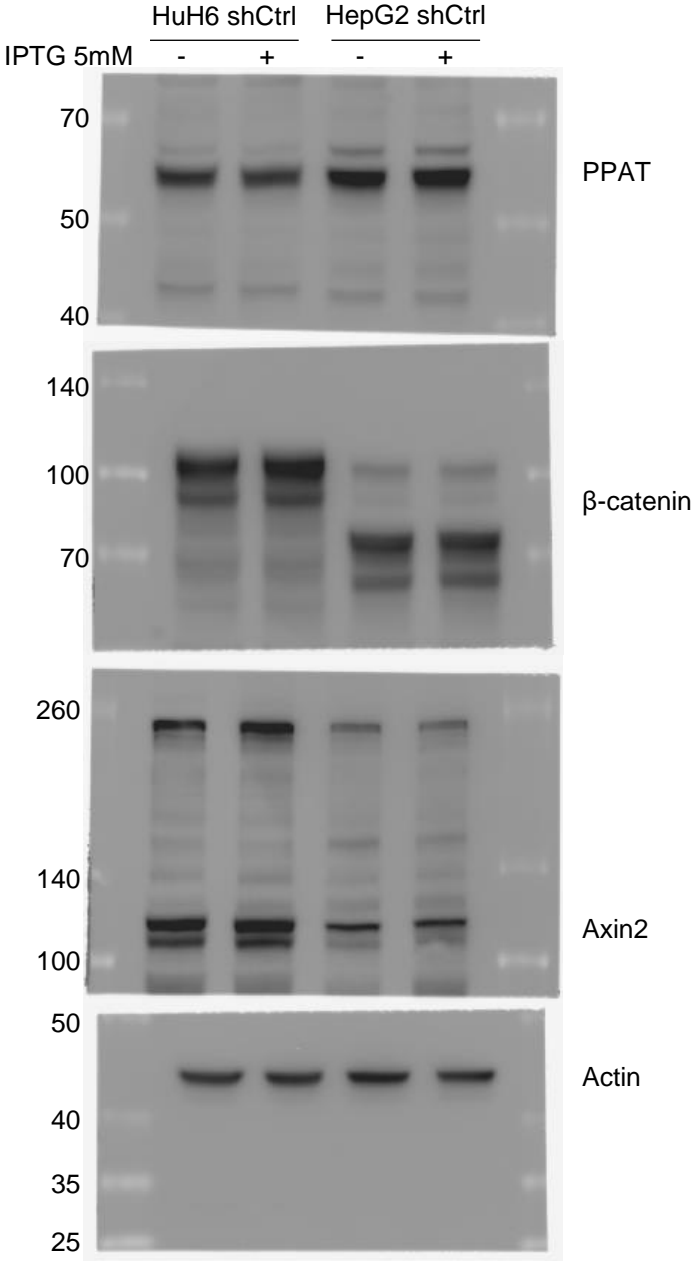

Supplement: Supplementary file 3 — Original western blots [file 41419_2025_7502_MOESM3_ESM.pdf]
